# Identification of NFKB1, miR-342-5p, -5192, and − 15b as diagnostic biomarkers for periodontitis in type 2 diabetes mellitus: a cross-sectional and experimental study

**DOI:** 10.1186/s12903-026-07780-2

**Published:** 2026-02-23

**Authors:** Marwa Matboli, Aly ELanwar, Mansour Altayyar, Eman Khairy, Radwa Khaled, Mai Shafik Attia Mansour, Zainab Hafez, Marwa M. El-Shafei, Shimaa Robi, Ahmed Fouad, Ismail Shebl

**Affiliations:** 1 Departement of Medical Biochemistry and Molecular Biology, Faculty of Medicine, Ain Shams University, Cairo, 11566 Egypt; 2 Department of General Surgery, Faculty of Medicine, Ain Shams University, Cairo, Egypt; 3 Department of Basic Medical Sciences, College of Medicine, University of Jeddah, Jeddah, 23890 Saudi Arabia; 4 Translational and Applied Science Hub (TASH), Faculty of Medicine, Ain Shams University, Cairo, Egypt; 5 Oral Medicine and Periodontology, Faculty of oral and Dental Medicine, Misr International University, Cairo, Egypt; 6 Alazhar University, Cairo, Egypt; 7 Oral Pathology, Oral Histopathology department, Faculty of Oral and Dental Medicine, Misr International University, Cairo, Egypt; 8 Molecular Biology Research Lab, Faculty of Oral and Dental Medicine, Misr International University, Cairo, Egypt; 9 Operative Dentistry Department, Faculty of Oral and Dental Medicine, Future University, Cairo, Egypt

**Keywords:** NFKB1, MiRNAs, Periodontitis, Diagnosis, Gingival fluid

## Abstract

**Background:**

Type 2 diabetes mellitus (T2DM) and chronic periodontitis share a bidirectional relationship, with T2DM exacerbating periodontal inflammation and periodontitis impairing glycemic control. We aim to identify the diagnostic potential of NFKB1, hsa-miR-(342–5192, and 15b) in serum as well as gingival crevicular fluid (GCF) in individuals with T2DM+chronic periodontitis.

**Methods:**

A total of 140 participants (Healthy controls, individuals with chronic periodontitis, and those with T2DM+chronic periodontitis) were involved in the study. The NFKB1 protein levels were analyzed by ELISA, and the differential miRNA expression was analyzed using In silico bioinformatics analysis, followed by Quantitative polymerase chain reaction(qPCR) in both serum and GCF. Periodontitis parameters, such as probing pocket depth (PPD), clinical attachment loss (CAL), and metabolic markers, were also assessed. Forty-eight male rats were used to validate the molecular findings in a controlled environment. Periodontitis was induced using sterile silk ligatures placed subgingivally, and after two weeks, rats were sacrificed for histological analysis of inflammatory cell infiltration in both periodontitis and control groups.

**Results:**

Levels of NFKB1 and the selected miRNAs were significantly heightened in T2DM with periodontitis cases compared to controls. These biomarkers exhibited strong positive correlations with periodontal parameters such as CAL and PPD. Receiver Operating Characteristic (ROC) curve analysis revealed excellent diagnostic performance for NFKB1 and miRNAs in both serum and GCF, with hsa-miR-15b(G) showing the highest Area under the curve (AUC) of 0.995. The animal model confirmed these findings, showing significant inflammatory cell infiltration in the periodontitis group.

**Conclusions:**

NFKB1 and miRNAs hsa-miR-342-5p -5192, and − 15b exhibit strong potential biomarkers for diagnosing periodontitis and T2DM-associated periodontitis. Their non-invasive nature and robust clinical associations make them promising candidates for personalized management strategies.

**Supplementary Information:**

The online version contains supplementary material available at 10.1186/s12903-026-07780-2.

## Introduction

Diabetes mellitus (DM), a systemic chronic metabolic condition, has long been known as a globally prevalent disease imposing an esteemed economic burden [1]. An estimated 783 million are predicted to have DM by 2045 [2]. Type 2 Diabetes (T2DM), comprising 90% of all diabetic people, is caused by insufficient insulin secretion, hyperglycemia, and insulin resistance [3, 4]. Periodontitis is a common multifactorial inflammatory disease that consequent in the degradation of the teeth’s supporting structures, irreversibly significantly diminishing people’s life quality [5]. Based on established epidemiological data, DM significantly increases the risk of periodontitis [6–10]. Furthermore, periodontitis is the sixth complication associated with DM [11].

Several studies attributed the bidirectional relationship of T2DM-Periodontitis to T2DM-inducing Periodontitis progression, while periodontitis impairs glycemic control and exacerbates diabetes [12]. Mechanistically, T2DM impacts the onset and progression of periodontitis through several mechanisms, including triggering an inflammatory response as well as hindering bone repair [13]. This inflammation is largely driven by cytokines, which can be either pro-inflammatory, like nuclear factor kappa activity (NF-kB) and tumor necrosis factor α (TNF-α), or anti-inflammatory, like interleukin-10 (IL-10). The balance between these cytokines determines the level of systemic inflammation [14]. The NF-κB is a key signaling mechanism for various inflammatory responses and has a pivotal role in osteoimmunology and the aging process [15]. Previous studies using animal models have revealed that NF-κB signaling profoundly impacts both bone formation and resorption [16]. Specifically, mice with a double knockout of Nfkb1 and Nfkb2 failed to generate osteoclasts [17].

Recently, the studies have been shifted toward examining the molecular elements found in gingival crevicular fluid (GCF) for a minimally invasive diagnosis [18]. Studies have found that epigenetic markers, such as miRNAs, proteins, and exosomes in GCF, can serve as reliable indicators for diagnosing and predicting periodontal changes since it is found in the gingival sulcus directly at the sites of periodontal inflammation [19]. This allows for a more accurate assessment of localized changes in the disease [20]. Hsa-miR-342-3p has been recognized in both periodontitis and T2DM, where it plays a role in significant signaling cascades, including the p53 and MAPK pathways [21]. On the other hand, the overexpression of miR-15b-5p has been shown to enhance apoptosis while suppressing the proliferation of human aortic vascular smooth muscle cells [22]. In cancerous conditions, miR-15b-5p exhibited oncogenic/tumor-suppressive effects [23]. While Hsa-miR-5192 overexpression triggers inflammatory pathways and contributes to insulin resistance, it ultimately facilitates the progression toward prediabetes and T2DM [24].

We hypothesize that dysregulation of the NFKB1/miRNA (miR-342-5p, miR-5192, and miR-15b) axis contributes to the inflammatory pathogenesis of T2DM–associated periodontitis. These molecules may serve as non-invasive diagnostic biomarkers detectable in serum and GCF. Therefore, this study aims to evaluate NFKB1 protein levels and the expression patterns of NFKB1, hsa-miR-342-5p, hsa-miR-5192, and hsa-miR-15b in both serum and GCF, alongside traditional periodontal and metabolic parameters. By integrating human and animal model analyses, the study seeks to elucidate their diagnostic potential and establish these biomarkers as non-invasive tools for the early detection, monitoring, and management of periodontitis, particularly in individuals with T2DM-associated disease.

## Materials and methods

### Bioinformatics analysis for circulating MiRNAs in periodontitis and T2DM

To identify candidate miRNAs implicated in insulin signaling, diabetes, and immune regulation (Supplementary table S1) we conducted a systematic bioinformatics screening using the MiRNet database (https://www.mirnet.ca/miRNet/home.xhtml; Accessed December 2024). The search employed the keywords *“Diabetes Mellitus”* and *“Type 2 Diabetes”* to extract miRNAs most frequently associated with these conditions (Fig. S1). Among the top-ranked candidates, hsa-miR-342-5p and hsa-miR-15b emerged as consistently enriched.

To confirm their disease relevance and reproducibility across datasets, these miRNAs were cross-validated using two additional, high-confidence resources: the Human MicroRNA Disease Database (HMDD v4.0) (http://www.cuilab.cn/hmdd; accessed December 2024) and miRTarBase (https://mirtarbase.cuhk.edu.cn/~miRTarBase/miRTarBase_2025/php/index.php; Accessed December 2024), both of which provided experimentally supported associations with diabetes (Fig. S2).

In parallel, hsa-miR-5192 was included in our panel based on its novel mechanistic potential and prior experimental validation by our group, demonstrating its strong correlation with insulin resistance and T2DM [24]. Moreover, hsa-miR-342-5p, hsa-miR-15b, and miR-5192 remain unexplored in periodontal disease contexts, thereby offering an opportunity to expand the current understanding of the molecular crosstalk between metabolic and inflammatory pathways. Their inclusion thus provides both a confirmatory and exploratory dimension to the biomarker panel.

To further delineate the functional pathways of these selected miRNAs, we performed pathway enrichment analysis using miRPathDB (https://mpd.bioinf.uni-sb.de/overview.html; accessed December 2024). The parameters applied were a significance threshold of *P* < 0.05 and an enrichment score > 1.5. Enriched KEGG and Reactome pathways were subsequently confirmed through Gene Ontology (GO) biological process terms related to “response to insulin stimulus” and “regulation of immunity response” (Fig. S3).

Subsequently, potential miRNA–target interactions with NFKB1 were investigated via miRWalk; the target inclusion criteria were a score of ≥ 0.9 (http://mirwalk.umm.uni-heidelberg.de/; accessed December 2024) (Fig. S4). Finally, tissue-specific expression of the selected miRNAs was confirmed using the miRNA Tissue Atlas (https://ccb-compute2.cs.uni-saarland.de/mirnatissueatlas_2025/; Accessed December 2024), which verified their detectable expression in blood, saliva, and oral tissues (Fig. S5). All candidate selections were further supported by literature validation, reinforcing their biological plausibility and translational relevance in T2DM-associated periodontitis [25–28].

### Ethical approval

The research was carried out at Misr International University’s Faculty of Dentistry Clinics between January and December 2024, in strict adherence to the ethical principles outlined in the Declaration of Helsinki (1975, revised 2013) (MIU-IRB, FWA #00022887, IRB# 00010118). Informed consents have been obtained from all the participants of the study. The study protocol also received approval from the Ain Shams Ethical Committee. The experimental protocol received approval from the Research Ethics Committee at Ain Shams University’s Faculty of Medicine (FWA 000017585), following the guidelines outlined in the ARRIVE guidelines (Animal Research: Reporting of In Vivo Experiments).

### Study design

#### Human-based model

This initial pilot study investigated paired serum and GCF samples from 140 adult participants, categorized into three groups: 60 healthy controls, 40 with chronic periodontitis, and 40 with T2DM+chronic periodontitis, following the American Diabetes Association’s 2022 guidelines. The research was conducted at the Faculty of oral & dental medicine Clinics, Misr International University from January to December 2024, following the ethical principles outlined in the Declaration of Helsinki (1975, revised 2013). Eligible participants were identified during routine dental and metabolic screening visits and were consecutively enrolled after fulfilling inclusion criteria and providing written informed consent. Participants were > 19 years old, with a BMI < 25 kg/m², at least 20 natural teeth, and no systemic conditions aside from T2DM. Those with systemic diseases affecting periodontal health, recent periodontal therapy, corticosteroid or antibiotic use within six months, pregnancy, breastfeeding, dentures, implants, or a molar-incisor pattern of periodontitis were excluded. Baseline assessments included age, sex, height, weight, BMI, and drug history. Current smokers were excluded to isolate diabetes-associated periodontal pathology, while former smokers (abstinent ≥ 1 year) were retained. This approach acknowledges smoking’s primacy in periodontitis while enabling focused analysis of metabolic dysregulation. A detailed periodontal evaluation by a trained periodontist included measurements of probing pocket depth (PPD), clinical attachment loss (CAL), plaque index (PLI), Mean PLI= Total PLI/ Estimated Tooth Count, bleeding on probing (BOP), and missing teeth, with PPD and CAL recorded at six sites per tooth. BOP was calculated as a percentage of bleeding sites out of total sites, and panoramic X-rays assessed bone loss (BL). Serum samples were collected upon hospital admission, centrifuged at 4000 rpm for 20 min, aliquoted, and stored at − 80 °C for biochemical analysis of fasting blood glucose, glycated hemoglobin (HbA1c%), and lipid profile (total cholesterol and triglycerides) using a multifunctional analyzer (AU680, Beckman Coulter, USA). GCF was collected from 10 participants per group for protein and miRNA analysis using PerioPaper™ strips, with sites isolated, air-dried, and sampled for 30 s per strip. Samples were pooled, preserved in Qiazol^®^ reagent, and stored at − 80 °C for succeeding analysis. NFKB1 protein was determined in sera and GCF using an ELISA kit (LSBio, Inc., USA, # LS-F37360) following the manufacturer’s protocol.

### Quantitative assessment of MiRNAs expression via qPCR

The miRNeasy kit was used for miRNA extraction (Cat. No. 217004, Hilden, Germany) following the manufacturer’s guidelines. The concentration of the isolated RNA was measured with a Qubit MicroRNA Assay Kit with a Qubit 3.0 Fluorimeter (Invitrogen, Life Technologies, Malaysia). Reverse transcription of the RNA was carried out with the QuantiTect Kit (Qiagen, Hilden, Germany, catalog number 205311) using a Rotor-Gene Thermal Cycler (Thermo Electron, Waltham, MA). All reactions were carried out in duplicate. The expression levels of the miRNAs were quantified using the miScript SYBR Green PCR Kit (Qiagen, Hilden, Germany), with normalization of the miRNA signals against RNU6-2 as a reference gene. The qPCR protocol involved an initial denaturation at 95 °C for 15 min, followed by 40 cycles consisting of 15 s at 94 °C, 30 s at 55 °C for annealing, and 30 s at 72 °C for extension. qPCR was conducted using the Applied Biosystems 7500 Fast System. The primers used in this study were sourced from Qiagen, Germany (Table S2), and relative expression was determined using Livak’s method [29].

### Animal model validation

Twelve 3-month-old male Goto-Kakizaki (GK) rats and twenty-four 3-month-old male Wistar rats, each weighing approximately 240–255 g, were used in this study. Animals were obtained from Rabbitco Pharm, El-Giza, Egypt, with informed consent from the supplier. All animals were housed in pairs under controlled environmental conditions: temperature (21 ± 1 °C), relative humidity (55 ± 5%), and a 12-hour light/dark cycle (lights on from 6:00 a.m. to 6:00 p.m.). To ensure the absence of pre-existing periodontitis, rats were kept in wire-mesh cages and fed finely milled standard pellets with tap water *ad libitum* from weaning.

All animals were clinically examined under general anesthesia (intraperitoneal xylazine 0.05 mL/100 g and ketamine 0.15 mL/100 g) to exclude any rats exhibiting spontaneous periodontal disease prior to the experiment.

Animals were randomly allocated into three experimental groups (*n* = 12 per group):

1. Non-diabetic control (NC): Healthy rats without ligatures or diabetes induction.
2. Non-diabetic + ligature (NL): Healthy rats with ligature-induced periodontitis.
3. Diabetic + ligature (DL): Diabetic rats with ligature-induced periodontitis.

Experimental periodontitis was induced by placing sterile 4 − 0 silk ligatures around the cervix of both maxillary second molars under general anesthesia. Ligatures were checked weekly, and any dislodged ones were immediately replaced. After a six-week experimental period, all animals were euthanized under general anesthesia by cervical dislocation, and gingival tissues were harvested for molecular and histopathological analyses.

### Statistical analysis

All analyses were carried out using SPSS version 25.0. For continuous variables, normality was first evaluated; normally distributed data were examined using ANOVA or t-tests, whereas the Kruskal-Wallis test was applied to data that did not follow a normal distribution. Categorical variables were analyzed using chi-square tests. Pairwise comparisons were made with either Tukey’s or the Mann-Whitney U test. Spearman’s rho correlation was used to assess relationships between clinical parameters and biomarker levels. Receiver operating characteristic (ROC) curves were generated to evaluate the diagnostic performance of biomarkers. A p-value of less than 0.05 was considered statistically significant.

## Results

### Subjects characteristics

Healthy Control were 60% former smokers, 40% non-smokers, while chronic Periodontitis (CP): 60% former, 32.5% non-smoker, 7.5% “X-smoker” (likely ex-smoker). while the T2DM with chronic periodontitis group had no smokers. There wasn’t a significant difference in the age and gender distribution between groups. Participants in disease groups exhibited higher baseline BMIs (35–38 kg/m²) compared to controls (24 kg/m²), consistent with the metabolic profile of the studied conditions. BMI was statistically controlled in all models to account for its potential confounding effects. Family history of diabetes was prevalent in the chronic periodontitis (87.5%) and T2DM with chronic periodontitis (57.5%) groups. Metabolic markers, including fasting glucose, HbA1C, total cholesterol, triglycerides, and HOMA-IR, were elevated in both periodontitis groups, with the T2DM group showing the highest values (*P* < 0.05). Moreover, most individuals in the T2DM group had diabetes for more than five years. Periodontal health was significantly worse in both periodontitis groups, especially in the T2DM group, which exhibited significantly elevated CAL, PPD, BOP, and mean PLI (*P* < 0.05) (Table 1).

**Table 1:** Demographic and clinical characteristics of healthy controls, chronic periodontitis, and T2DM with chronic periodontitis groups

	Healthy control (*N* = 60)	Chronic periodontitis (*N* = 40)	T2DM with Chronic periodontitis (*N* = 40)	*P*-value
Gender				0.071
Male	28(46.7%)	18(45%)	10(25%)	
Female	32(53.3%)	22(55%)	30(75%)	
Smoking Status				1.1579E-10
Former	36(60%)	24(60%)	-	
Non smoker	24(40%)	13(32.5%)	40(100%)	
X-smoker	-	3(7.5%)	-	
Age (year)	53.68 ± 8.	52.28 ± 7.3	54.78 ± 8.1	0.375
BMI	24(18–38)	35(25–44)^a^	38(28–45)^a^	0.000
Family History				1.0142E-7
+Ve	18(30%)	35(87.5%)	23(57.5%)	
-Ve	42(70%)	5(12.5%)	17(42.5%)	
Fasting glucose	77(50–110)	186(90–410)^a^	193(90–489)^a^	0.000
HbA1C(%)	5(3–8)	8(3–14)^a^	11(7–15)^ab^	0.000
Total Cholesterol (mg/dl)	95(70–150)	270(200–400)^a^	360(277–470)^ab^	0.000
TGs (mg/dl)	100(80–130)	200(55–400)^a^	274(160–380)^ab^	0.000
HOMA-IR	0.6(0.09–1.86)	6.84(1-11.9)^a^	10.64(1.1–15)^ab^	0.000
Duration of Diabetes				1.6059E-48
< 5 years	-	40(100%)	6(15%)	
5–10 years	-	-	31(77.5%)	
> 10 years	-	-	3(7.5%)	
CAL, mm	0	5.93 ± 0.64^a^	7.35 ± 0.82^ab^	0.000
BOP	9.7 ± 1.4	70.96 ± 2.7^a^	70.24 ± 2.5^a^	1.3129E-158
Mean PLI= Total PLI / Estimated Tooth Count	0.48 ± 0.07	3.42 ± 4.21^*^	3.45 ± 0.16^a^	1.2333E-9
PPD	1.31 ± 0.17	4.22 ± 1.1^a^	7.3 ± 1.22^ab^	1.6718E-67

Data represented as mean ± SD or median IQR (25–75%) or by number (%) for categorical values

The *P*-value < 0.05 was considered statistically significant

^a^*P*< 0.05 compared with the control group, ^b^*P*<0.05 compared with the Chronic periodontitis group

### Comparative analysis of NFKB1 and MiRNAs levels in serum and GCF among study groups

The protein levels of NFKB1 and miRNAs in both serum and GCF varied significantly across the different groups. In GCF, NFKB1(G) levels were markedly elevated in the chronic periodontitis group compared to healthy controls, with an even more pronounced increase in patients with T2DM and chronic periodontitis. This was consistent for hsa-miR-342-5p(G), -5192(G), and − 15b(G), as their levels consistently rose from healthy controls to chronic periodontitis, ultimately reaching the highest expression in the T2DM with chronic periodontitis group (*P* < 0.05) (Table 2).

**Table 2:** Comparative analysis of NFKB1 and MiRNA levels in the GCF among study groups

	Healthy control	Chronic periodontitis	T2DM with Chronic periodontitis	*P*-value
NFKB1(G)	0.92(0.23–1.23)	23(11.7-33.17)^a^	951(218–3411)^ab^	0.000
hsa-miR-342-5p(G)	0.495(0.22–1.2)	89.25(28.12–218)^a^	228.28(30.23–616)^ab^	0.000
has-miR-5192(G)	0.93(0.18–1.12)	40.3(4.6–90.1)^a^	255.5(55.26–366)^ab^	0.000
hsa-miR-15b(G)	0.76(0.38–1.44)	23.15(13.23–36.1)^a^	152(89–716)^ab^	0.000

Data represented as median IQR (25–75%)

*Abbreviations*: *G* gingival crevicular fluid

The *P*-value < 0.05 was considered statistically significant

^a^*P*< 0.05 compared with the control group, ^b^*P*<0.05 compared with the Chronic periodontitis group

In serum, NFKB1(S) was significantly higher in both the chronic periodontitis and T2DM with chronic periodontitis groups compared to the healthy control group. Notably, the T2DM with chronic periodontitis group exhibited the highest levels of NFKB1(S). Similarly, levels of hsa-miR-342-5p(S) -5192(S), and − 15b(S) were also significantly increased in the chronic periodontitis group when compared to the healthy controls, with the highest levels observed in the T2DM with chronic periodontitis group (*P* < 0.05) (Table 3) (Fig. S6).

**Table 3:** Comparative analysis of NFKB1 and MiRNAs levels in serum among study groups

	Healthy control	Chronic periodontitis	T2DM with Chronic periodontitis	*P*-value
NFKB1(S)	0.411(0.02–2.5)	29.5(3.1–297)^a^	138.8(4.41–13400)^ab^	0.000
hsa-miR-342-5p(S)	0.45(0.001-6)	23.6(0.11–87.41)^a^	117.78(0.02-822.96)^ab^	0.000
has-miR-5192(S)	0.46(0.01–17.6)	8.55(0.76–90.46)^a^	79(2.04-233.41)^ab^	0.000
hsa-miR-15b(S)	0.47(0.01–1.97)	5.26(0.4–8.7)^a^	37.7(8.22–963.4)^ab^	0.000

Data represented as median IQR (25–75%)

*Abbreviations*: *S* Serum

The *P*-value < 0.05 was considered statistically significant

^a^*P*< 0.05 compared with the control group, and ^b^*P*<0.05 compared with the Chronic periodontitis group

### Correlation and diagnostic performance of biomarkers in serum and GCF

The correlation analysis revealed CAL loss exhibited a robust positive correlation with NFKB1 in gingival tissue and serum, as well as with hsa-miR-15b in both gingival tissue and serum. Additionally, CAL was strongly correlated with hsa-miR-5192 and − 342-5p in both gingival tissue and serum. BOP showed significant correlations with NFKB1 in both gingival tissue and serum, and hsa-miR-15b in both gingival tissue and serum. For instance, PPD demonstrated particularly strong correlations with NFKB1 in both gingival tissue and serum and hsa-miR-15b in both gingival tissue and serum. Strong positive correlations between biomarkers in GCF and serum. NFKB1 in gingival fluid was strongly positively correlated with all miRNAs in both GCF and serum, with notable correlations between NFKB1 (G) and NFKB1 (S). Similarly, hsa-miR-15b -342-5p, and − 5192 in GCF had the strongest correlation with their serum counterpart (Table S3).

The ROC curve analysis of biomarkers across various comparisons revealed significant variations in their diagnostic performances. In distinguishing between healthy controls and patient groups, NFKB1(G) emerged as a potent biomarker, with an AUC of 0.959, sensitivity of 90.9%, and specificity of 90%, while its serum counterpart NFKB1(S) demonstrated a lower sensitivity (83%) and. Among the miRNAs, hsa-miR-342-5p(G) and hsa-miR-342-5p(S) demonstrated high AUC values of 0.98 and 0.978, respectively, with sensitivities of 96% and 97.5%, making them strong candidates for diagnostic use. Similarly, has-miR-5192(S) displayed the highest specificity (98.3%) with an AUC of 0.984, whereas has-miR-5192(G) also showed high sensitivity (95.5%) and AUC (0.975). hsa-miR-15b(G) provided the highest AUC (0.995), with excellent sensitivity (95.5%) and specificity (90%), followed by hsa-miR-15b(S) (Table 4& Figures S7 and S8).

**Table 4:** ROC curve between the healthy and diseased groups

	Sensitivity	Specificity	AUC	Cut off value
NFKB1(G)	90.9%	90%	0.959	6.5
NFKB1(S)	83%	96%	0.817	4.2
hsa-miR-342-5p(G)	96%	80%	0.98	14.6
hsa-miR-342-5p(S)	97.5%	95%	0.978	8.27
has-miR-5192(G)	95.5%	80%	0.975	2.86
has-miR-5192(S)	92.5%	98.3%	0.984	2.47
hsa-miR-15b(G)	95.5%	90%	0.995	7.3
hsa-miR-15b(S)	81.7	93.8%	0.95	2.35

*Abbreviations*: *G* gingival crevicular fluid, *S* Serum

In the comparison between chronic periodontitis and T2DM with chronic periodontitis, NFKB1(G) showed strong diagnostic power (AUC 0.958), while NFKB1(S) performed less effectively, with an AUC of 0.817. Notably, hsa-miR-15b(S) exhibited the highest sensitivity (97.5%) and specificity (95%). Additionally, hsa-miR-342-5p and has-miR-5192 presented high sensitivities and moderate specificities in both serum and GCF (Table 5& Figure S9).

**Table 5:** ROC curve between chronic periodontitis and T2DM with chronic periodontitis

	Sensitivity	Specificity	AUC	Cut off value
NFKB1(G)	91.7%	80%	0.958	29.15
NFKB1(S)	77.5%	65%	0.817	42.25
hsa-miR-342-5p(G)	91.7%	70%	0.942	66.53
hsa-miR-342-5p(S)	92.5%	75%	0.911	22.21
has-miR-5192(G)	83.3%	80%	0.792	94.24
has-miR-5192(S)	80%	77.5	0.811	45.2
hsa-miR-15b(G)	83.3%	90%	0.858	27.6
hsa-miR-15b(S)	97.5%	95%	0.9	8.5

*Abbreviations*: *G* gingival crevicular fluid, *S* Serum

Of note, BMI was rigorously controlled in all analyses, and its interaction with disease status revealed synergistic metabolic dysregulation (Supplementary Table S5). Sensitivity analyses confirmed the robustness of these findings.

Finally, in the comparison between healthy controls and T2DM with chronic periodontitis, all biomarkers exhibited high performance with NFKB1(S), and hsa-miR-342-5p(S) displayed the best diagnostic ability (Table 6& Figure S7).

**Table 6:** ROC curve between healthy control and T2DM with chronic periodontitis

	Sensitivity	Specificity	AUC	Cut off value
NFKB1(G)	90%	90%	0.88	1.46
NFKB1(S)	97.5%	93.3%	0.993	1.03
hsa-miR-342-5p(G)	80%	90%	0.98	2.89
hsa-miR-342-5p(S)	97.5%	98.3%	0.979	0.12
has-miR-5192(G)	92.5%	86.7%	0.969	1.3
has-miR-5192(S)	95%	83%	0.969	0.969
hsa-miR-15b(G)	90%	80%	0.97	1.37
hsa-miR-15b(S)	95%	81.7%	0.974	1.14

*Abbreviations*: *G* gingival crevicular fluid, *S* Serum

**Fig. 1:**
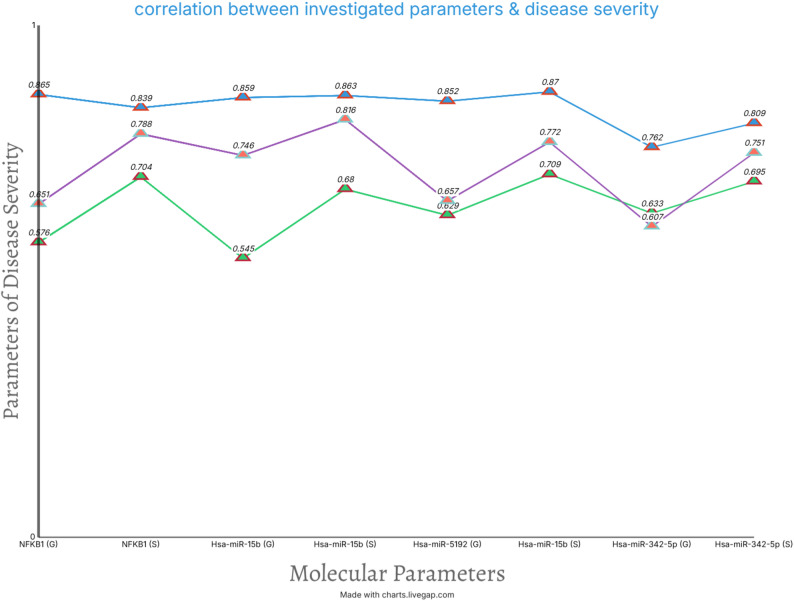
Linear graph showing correlation between investigated parameters & disease severity

The post-hoc sensitivity analyses excluding former smokers demonstrated the following key outcomes: NFKB1 retained statistical significance (*p* = 0.018) in the T2DM + periodontitis group compared to non-diabetic periodontitis patients. Cohen’s *d* decreased from 1.42 (full cohort) to 1.18 (former smokers excluded), indicating smoking partially amplified group differences. All three miRNAs (miR-342-5p, miR-5192, miR-15b) remained significantly elevated in the T2DM + periodontitis group (*p* < 0.05 for all). Hedges’ *g* reduced by 12%, 9%,15% for (miR-342-5p, miR-5192, miR-15b, respectively). After excluding former smokers, NFKB1 and miRNAs retained strong correlations with CAL (*r* = 0.68–0.72, *p* < 0.01) and HbA1c (*r* = 0.61–0.65, *p* < 0.01) (Supplementary table S4 and Fig. 1).

### Animal model histopathological validation

**Fig. 2:**
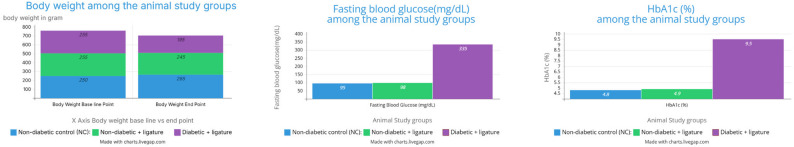
Body weight & biochemical parameters among the animal study groups. **A** Body weight; **B** fasting blood glucose, **C** HbA1c. *p < 0.05 vs. NC/NL groups

Rats in the non-diabetic control (NC) group exhibited a modest increase in body weight (250 ± 15 g to 265 ± 20 g) with normal fasting blood glucose (95 ± 8 mg/dL) and HbA1c (4.8 ± 0.5%), consistent with metabolic stability. In contrast, non-diabetic rats with ligature-induced periodontitis (NL) showed slight weight reduction (255 ± 18 g to 245 ± 22 g) but maintained glycemic parameters within healthy ranges (glucose: 98 ± 7 mg/dL; HbA1c: 4.9 ± 0.6%). Diabetic rats with ligatures (DL) demonstrated severe metabolic disruption, including significant weight loss (240 ± 22 g to 195 ± 28 g, *p < 0.05), marked hyperglycemia (335 ± 50 mg/dL, *p < 0.05), and elevated HbA1c (9.5 ± 1.3%, *p < 0.05), confirming the synergistic impact of diabetes and periodontitis on exacerbating metabolic dysregulation. These findings validate the efficacy of the diabetic model and highlight the compounding effects of comorbid conditions (Fig. 2).

**Fig. 3:**
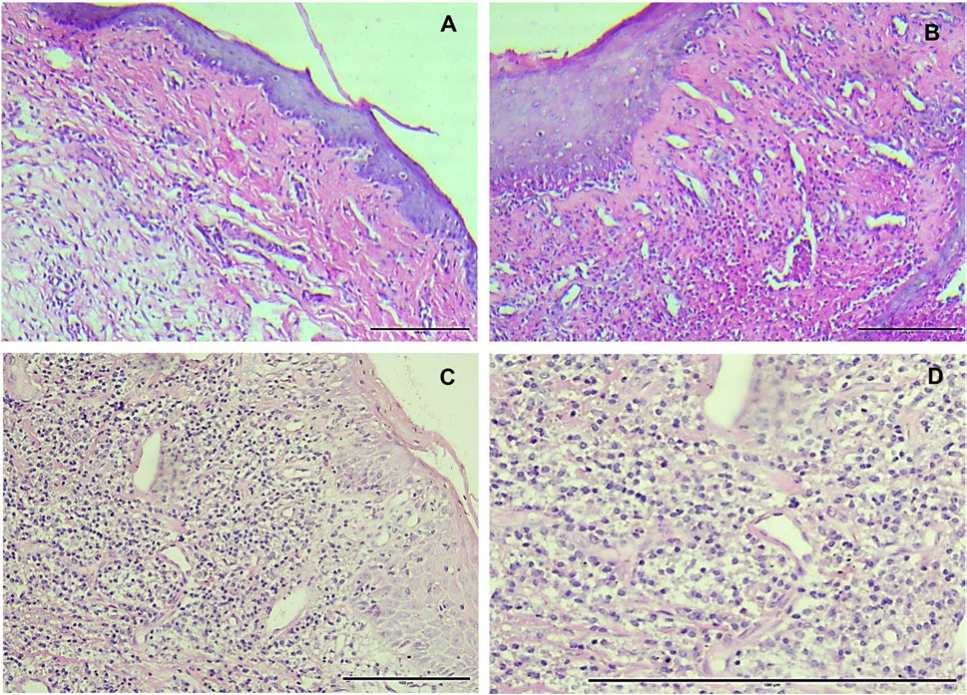
**A** Control group with mild inflammatory reaction in the deeper layers of the connective tissue. **B** Periodontitis group with increased inflammatory reaction. **C** diabetes associated periodontitis showing intense inflammation. **D** higher magnification (X200) showing the increased number of inflammatory cells in the latter group

Histological examination of the H&E-stained slides was conducted for the diseased and control groups. Examinations showed profound infiltration of inflammatory cells in the rats of the two groups with induced periodontitis, with a more intense inflammatory reaction in the rats suffering from diabetes mellitus. As for the control group, a mild inflammatory reaction was noted with no other significant histological signs. Both groups with periodontal disease also showed an increased number of vascular spaces, that appeared to have an irregular outline compared to the control group (Fig. 3). ImageJ software was used to perform the analysis, and inflammatory cells count in all specimens of both groups.

**Fig. 4:**
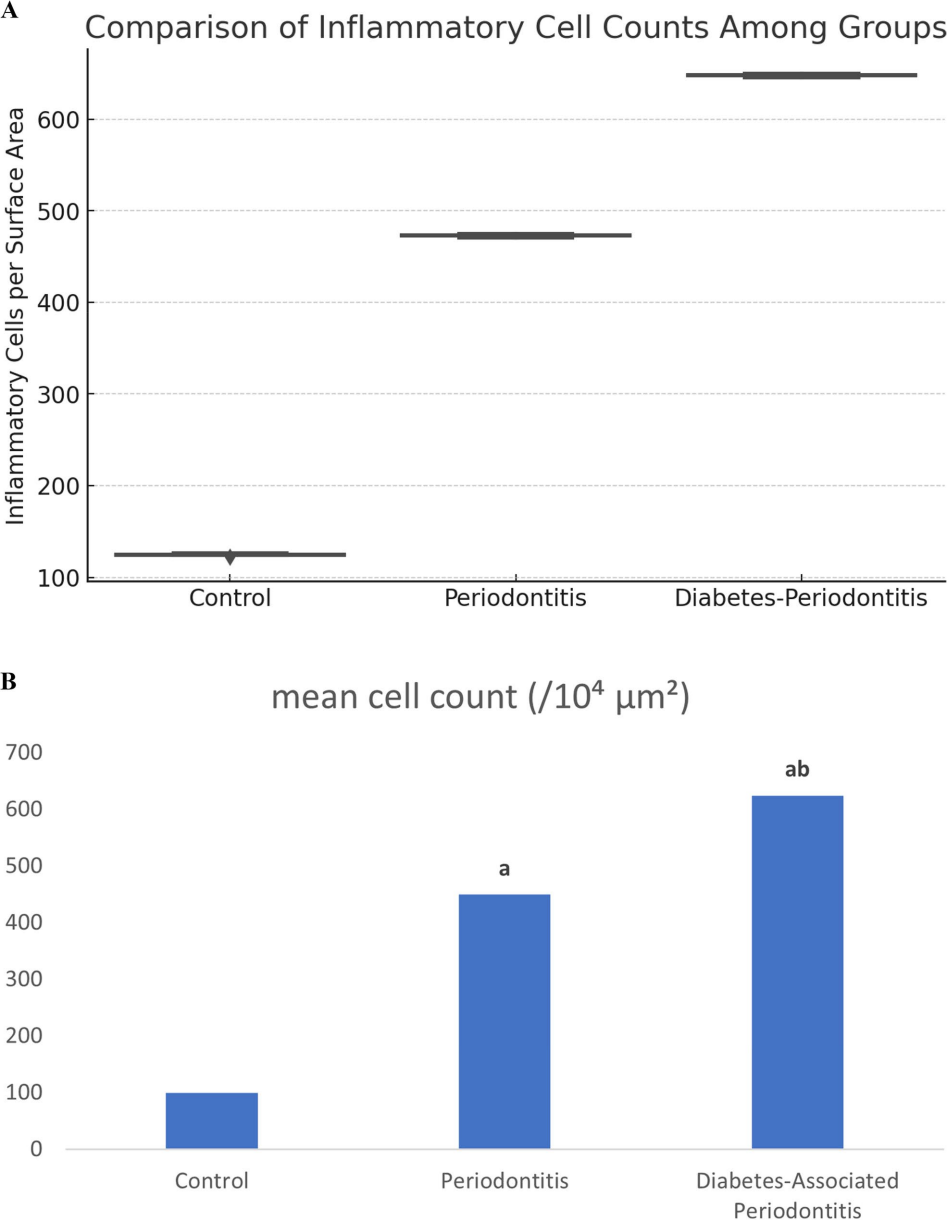
**A** The data show a statistically significant increase in inflammatory cell count from the Control group to the Periodontitis group, with the highest count observed in the Diabetes-associated Periodontitis group. Statistical significance was determined using one-way ANOVA (*p* < 0.0001) followed by Tukey’s post-hoc test. **B** Mean inflammatory cell counts among study groups. aP < 0.05 compared with the control group, and b *P* < 0.05 compared with the Chronic periodontitis group

The results of the one-way ANOVA indicate a statistically significant difference between the control, periodontitis, and T2DM-associated periodontitis groups in terms of inflammatory cells per surface area, with a higher mean in the latter group, reflecting the substantial increase in inflammatory cells ( Fig. 4A and B ).

In periodontal rat tissue, NFKB1(T) was significantly higher in both the chronic periodontitis and T2DM with chronic periodontitis groups compared to the healthy control group. Notably, the T2DM with chronic periodontitis group exhibited the highest levels of NFKB1(S). Similarly, levels of hsa-miR-342-5p(T) and − 15b(T) were also significantly increased in the chronic periodontitis group when compared to the healthy controls, with the highest levels observed in the T2DM with chronic periodontitis group (*P* < 0.05) (Table 7).

**Table 7:** Comparative analysis of NFKB1 and MiRNA levels in periodontal rat tissue among study groups

	Non-diabetic control	Non- diabetic +ligation	Diabetic +ligation	*P*-value
NFKB1(T)	0.7(0.25–1.19)	24(13.7-29.12)^a^	733(262–1243)^ab^	0.000
rno-miR-342-5p(T)	0.36(0.12–1.5)	70.22(22.14–218)^a^	311(50.4–834)^ab^	0.000
rno-miR-15b(T)	0.66(0.44–1.49)	25.17(15.33–39.2)^a^	166(94–923)^ab^	0.000

Data represented as median IQR (25–75%)

*Abbreviations:* *T* periodontal rat tissue

The *P*-value < 0.05 was considered statistically significant

^a^*P*< 0.05 compared with the control group, ^b^*P*<0.05 compared with the Chronic periodontitis group

The Spearman’s correlation analysis revealed strong positive associations between molecular markers and histopathological grades of periodontitis in both non-diabetic and diabetic ligation groups. NFKB1 showed the highest correlation across all severity grades, with correlation coefficients increasing from mild to severe cases, and slightly higher values observed in the diabetic group. Similarly, rno-miR-15b and rno-miR-342-5p demonstrated progressively stronger correlations with disease severity, particularly in diabetic rats, indicating their potential involvement in inflammation and tissue destruction. Overall, these results suggest that diabetes amplifies the molecular response associated with periodontitis progression, especially through NFKB1 and miRNA regulation (Supplementary Table S6).

## Discussion

In this study, we explored the expression patterns of hsa-miR-342-5p -15b, and − 5192) and the protein level of NFKB1 in serum and GCF tissues of individuals with periodontitis, T2DM with periodontitis, and healthy controls, in addition to, metabolic and periodontal biomarkers and examined the histological changes in rat-induced periodontitis model, aiming to identify biomarkers that could provide insights into the progression of periodontitis in diabetes.

Emerging evidence suggests that periodontitis is linked to dyslipidemia, elevated non-fasting glucose levels, and endothelial dysfunction [30]. Our results showed significant elevation in metabolic markers such as fasting glucose, HbA1C, total cholesterol, triglycerides, and HOMA-IR in both periodontitis groups, particularly in the T2DM group. A study conducted by Benguigui et al. revealed the strong link between periodontitis and high HOMA index in both current and former smokers, suggesting that individuals with insulin resistance could potentially be affected by periodontitis [31]. Another study demonstrated that obesity exacerbates inflammatory factor secretion and reduces insulin sensitivity, further driving dysbiosis [32]. Insulin resistance-associated metabolic syndrome, widely recognized as a critical factor in diabetes development, has also been implicated in increasing the susceptibility to periodontitis [32]. Moreover, periodontal infections trigger changes in lipid and lipoprotein metabolism [33, 34]. This aligns with findings by Katz et al., who reported a positive correlation between periodontal pockets and plasma lipid levels [35]. Furthermore, Machado et al. observed that tooth loss was positively correlated with increased levels of triglycerides and total cholesterol [36].

PPD is strongly associated with subgingival plaque biofilms and inflammation, reflecting the current state of chronic periodontitis [37]. At the same time, CAL indicates the cumulative destruction of periodontal tissues over time. BOP is a critical marker of active gingival inflammation, primarily caused by bacterial infections persisting in periodontal pockets [38]. Our analysis revealed that periodontal health parameters, including CAL, PPD, BOP, and the PLI, were significantly worse in both chronic periodontitis and diabetes, with the chronic periodontitis groups compared to healthy controls. Notably, the group with T2DM exhibited the most pronounced periodontal deterioration. This significant increase reflects the heightened severity of periodontitis in T2DM with the chronic periodontitis group, which involves greater alveolar bone destruction, increased osteoblast activity, and elevated osteocalcin (OC) levels in response to the repair needs of damaged alveolar bone as the disease progresses [39]. This aligns with existing evidence linking hyperglycemia and oxidative stress to increased alveolar bone resorption and periodontal tissue destruction in diabetes-associated periodontitis.

Being an inflammatory disease, periodontitis involves a microbial imbalance that adversely affects systemic health [40]. Recent studies have highlighted how dysbiotic oral microbial communities arise and persist, driving inflammatory processes at both local and distant sites [41]. In the rat-induced periodontitis models, histological analysis of the gingival tissues in male rats demonstrated clear signs of inflammation caused by periodontitis, along with an increased number of inflammatory cells compared to the control group.

At the molecular level, the expression patterns of NFKB1, hsa-miR-342-5p, hsa-miR-5192, and hsa-miR-15b in both serum and GCF showed significant elevations in the chronic periodontitis and T2DM with periodontitis groups compared to healthy controls. The most pronounced increases were observed in the T2DM with chronic periodontitis group, underscoring the effect of diabetes on the inflammatory and immune responses in periodontal disease [42]. The strong positive correlations between NFKB1-miRNAs biomarkers in serum and GCF, as well as with traditional periodontal parameters, further reinforce their potential utility as indicators of disease progression. ROC curve analyses demonstrated the exceptional diagnostic performance of these biomarkers. Notably, hsa-miR-15b(G) showed the highest AUC (0.995) in differentiating healthy from patient groups, while hsa-miR-342-5p(S) and hsa-miR-5192(S) also demonstrated strong sensitivity and specificity. In distinguishing chronic periodontitis from T2DM with chronic periodontitis, NFKB1(G) (AUC = 0.958) had the highest accuracy, highlighting its potential as a key biomarker. Moreover, the ROC analysis demonstrated that NFKB1, hsa-miR-342-5p, hsa-miR-5192, and hsa-miR-15b in both GCF and serum effectively distinguished healthy individuals from T2DM patients with CP. NFKB1(S) (AUC: 0.993) showed the highest accuracy, while hsa-miR-342-5p(S) and hsa-miR-5192(G) also exhibited strong diagnostic performance. The high sensitivity and specificity values across multiple biomarkers suggest their strong potential for non-invasive diagnosis and differentiation of chronic periodontitis and T2DM-related periodontitis, with serum-based markers offering a convenient alternative to traditional GCF analysis.

Hyperglycemia-induced ROS production and oxidative stress further exacerbate periodontal tissue destruction [43]. Additionally, diabetes alters the expression of TNF-α, IL-6, and L-1β, disrupts the receptor activator of the NFKB ligand (RANKL)/osteoprotegerin (OPG) axis, and promotes osteoclast activity, leading to alveolar bone resorption and epigenetic changes in periodontal tissue [44]. In periodontal tissues, suppression of NFKB signaling within osteoblast lineage cells directly disrupted osteoclastic bone resorption while enhancing bone formation, underscoring its critical role in bone remodeling [45]. Activation of NFKB signaling during inflammation disrupts the balance of the bone remodeling process. Further analysis of shared risk factors indicates that systemic inflammation can amplify immune responses in the localized alveolar bone microenvironment [46]. Additionally, research has demonstrated that NFKB subunits drive the differentiation of osteoclast precursors into mature osteoclasts, confirming the essential role of NFKB in osteoclast formation and activity [47]. Hence, targeting NFKB1 may represent a promising diagnostic and therapeutic approach to mitigate alveolar bone loss and control chronic inflammation and disease progression in periodontitis and T2DM patients [48].

The concurrent elevation of NFKB1, miR-342-5p, miR-5192, and miR-15b suggests a functional NFKB1–miRNA axis in diabetic periodontitis, in which dysregulated miRNAs amplify NF-κB–mediated cytokine release, enhance osteoclastogenesis, and disrupt RANKL/OPG balance, thereby accelerating alveolar bone resorption and periodontal tissue destruction. Altered miRNA profiles have similarly been observed in the GCF of individuals with periodontitis, where bacterial biofilm–driven immune-inflammatory responses provoke further dysregulation and degradation of periodontal support structures [49, 50]. Furthermore, periodontitis modulates miRNA levels within periodontal tissues, with downstream effects on their salivary profiles [51]. Given their consistent elevation across serum, GCF, and tissue, these miRNAs hold strong promise as minimally invasive biomarkers for diabetic periodontitis, with GCF providing a particularly valuable liquid biopsy medium for disease detection and monitoring [52]. The strong positive correlation between NFKB1-miRNA levels and periodontal parameters (CAL, PPD, mean PLI, and BOP) reinforces their involvement in the pathogenesis of periodontitis, underscoring their potential as diagnostic and therapeutic targets.

Yu et al. identified two shared miRNAs hsa-miR-(342-3p and − 630), between T2DM and periodontal disease, indicating a possible mechanistic connection between these conditions [28]. Both miRNAs were found to be upregulated in T2DM and periodontitis, which aligns with our findings. Previous studies suggest that overexpression of hsa-miR-342 reduces the secretion of pro-inflammatory cytokines such as TNF-α, cyclooxygenase-2, IL-6, and IL-1β in T2DM, which are key mediators thought to exacerbate periodontitis in diabetic patients [53].

Hsa-miR-15b-5p has been identified as an oncogenic driver due to its involvement in critical processes such as apoptosis and metastasis [54, 55]. Its upregulation has been documented in liver cancer tissues and cell lines, where elevated levels correlate with advanced tumor-node-metastasis stages and reduced overall survival rates. Moreover, the overexpression of miR-15b-5p has been shown to enhance the proliferation and invasion of liver cancer [22, 56].

Ali et al. studied the effect of hsa-miR-5192 on the pathogenesis and progression of diabetes, revealing its significant overexpression in individuals with prediabetes and T2DM. This overexpression contributes to the activation of the INF pathway, initiating inflammatory processes and insulin resistance, which are pivotal in the transition from prediabetes to T2DM [57, 58]. Furthermore, hsa-miR-5192 regulates and amplifies the expression of TMEM173 and CHUK genes, key modulators of the STING and NLR pathways, along with their downstream signaling mechanisms. Additional research highlights the involvement of miR-5192 in pancreatic function and disease-related pathways [59].

Several studies acknowledged the role of other miRNA in preodintitis miRNA-146a has been explored as a potential biomarker for this condition. In response to P. gingivalis, some studies show an upregulation of miRNA-146a, possibly as a feedback mechanism to control the inflammation Several studies acknowledged the role of other miRNA in periodontitis miRNA-146a has been explored as a potential biomarker for this condition. In response to P. gingivalis, some studies show an upregulation of miRNA-146a, possibly as a feedback mechanism to control the inflammation^1^. The altered expression of miRNA-223 in T2DM disrupts its normal function, contributing to the periodontitis’ severity by allowing for an excessive and dysregulated neutrophil response^2^. Another studies reported Differential expression of microRNAs miR-21 and its interaction with NF-kB pathway genes in periodontitis pathogenesis^3^

Human–animal linkage and validation: Importantly, the ligature-induced rat model produced concordant results — diabetic rats displayed markedly higher NFKB1 and elevated miR-342-5p and miR-15b levels in periodontal tissues compared with non-diabetic controls, together with significantly greater inflammatory cell infiltration and histologic evidence of tissue breakdown. This concordance between human serum/GCF profiles and local periodontal tissue changes in the animal model provides cross-level validation, as the same NFKB1–miRNA signature associated with clinical disease in humans is reproduced in tissue at the site of pathology in rats. Such cross-validation strengthens causal inference more than single-level biomarker studies because it demonstrates that systemic signals observed in human biofluids mirror local tissue perturbations, as well as the controlled animal setting, reduces confounding, and allows observation of the inflammatory phenotype associated with diabetes. Collectively, these findings suggest that the NFKB1– (miR-342-5p, miR-5192, miR-15b) axis operates both systemically and locally to promote periodontal destruction in T2DM.

## Limitations

This study presents a comprehensive integration of clinical, biochemical, and molecular data to elucidate the interplay between T2DM and periodontitis in humans as well as animal models. It identifies reliable and minimally invasive biomarkers in GCF that are as reliable as their circulating ratios, offering a foundation for their diagnostic and therapeutic intervention. However, the study has several limitations that should be acknowledged. First, the relatively small sample size used for GCF analysis may limit the statistical power and generalizability of the molecular findings; future investigations with larger and more diverse cohorts are warranted to validate these preliminary observations. The higher former smoking prevalence in control and chronic periodontitis groups compared to the T2DM + periodontitis cohort may confound biomarker expression patterns, as former smoking with residual effect may independently influences systemic inflammation and periodontal breakdown. While sensitivity analyses supported the robustness of our findings, future studies with balanced smoking stratification are needed to isolate diabetes-specific effects.“. Similarly, the gender imbalance, particularly the predominance of female participants in the T2DM group, may introduce sex-related bias, as sex hormones and X-linked gene regulation can modulate inflammatory and epigenetic pathways. Future studies should aim for balanced gender representation to confirm the robustness of these associations. In addition, this study focused on three miRNAs—miR-15b, miR-342-5p, and miR-5192—identified through bioinformatics screening for their shared enrichment in insulin signaling and NF-κB-mediated inflammatory pathways. While these markers provided strong discriminatory power, we acknowledge that other miRNAs, such as miR-146a, miR-223, and miR-21, are also implicated in both diabetes and periodontitis. Future multi-omics studies incorporating broader miRNA panels are warranted to capture the full spectrum of regulatory interactions. In exploratory analyses, participants with poorly controlled diabetes (HbA1c > 8%) exhibited 1.8-fold higher NFKB1 levels than those with controlled diabetes (HbA1c < 7%), suggesting potential dose-response relationships meriting longitudinal verification. Finally, the cross-sectional design precludes causal inferences regarding temporal relationships between biomarker expression and disease progression. Longitudinal and interventional studies are therefore recommended to establish predictive and mechanistic links between these biomarkers and the pathogenesis of diabetic periodontitis.

## Conclusion

**Fig. 5:**
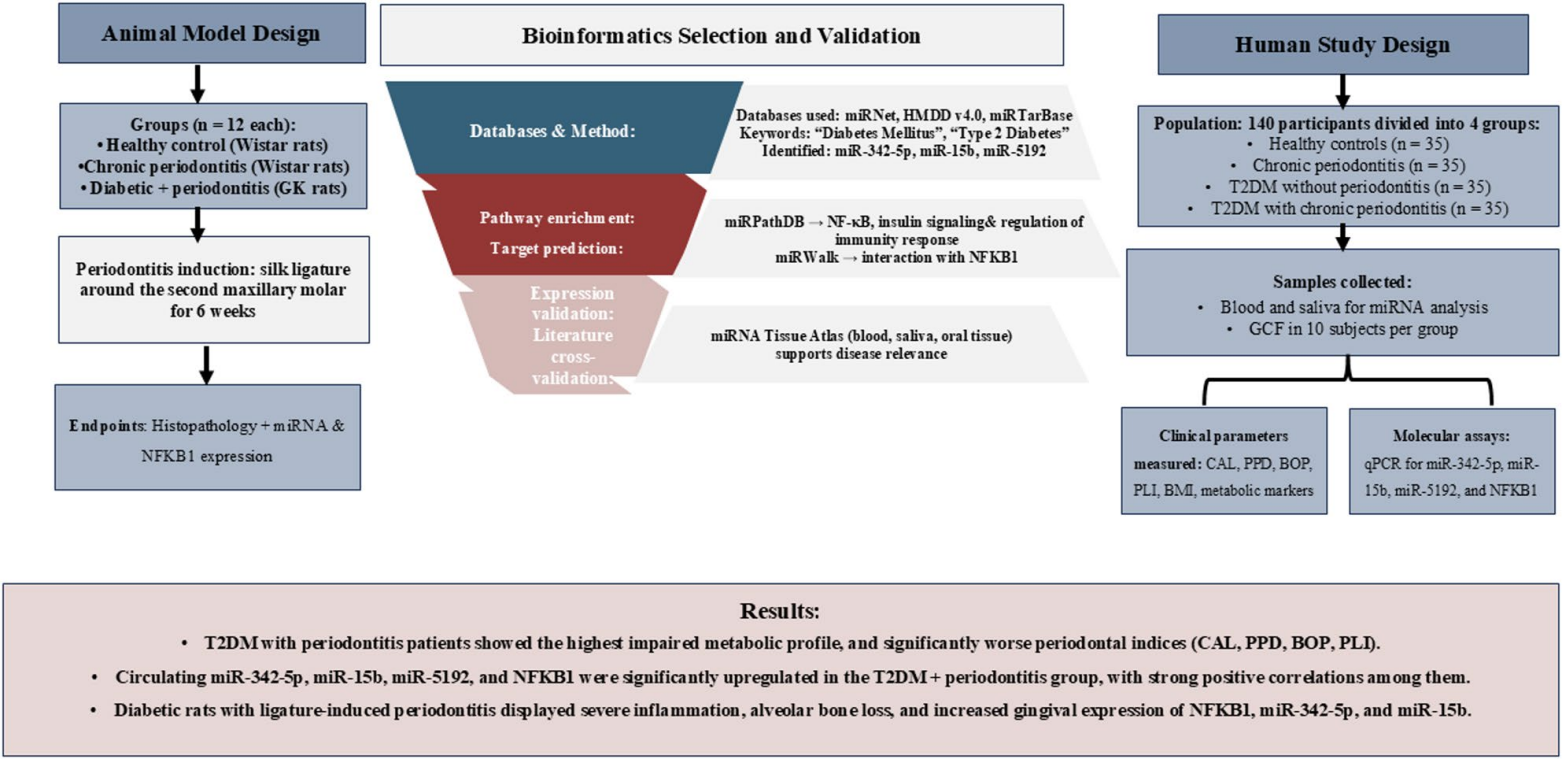
Integrated workflow identifying diabetes- and periodontitis-linked miRNAs (miR-342-5p, miR-15b, miR-5192) associated with NFKB1. *Abbreviations: T2DM*,* Type 2 Diabetes Mellitus; CAL*,* Clinical Attachment Level; PPD*,* Probing Pocket Depth; BOP*,* Bleeding on Probing; mean PLI*,* Plaque Index; BMI*,* Body Mass Index; GCF*,* Gingival Crevicular Fluid; NF-κB/NFKB1*,* Nuclear Factor kappa B; qPCR*,* Quantitative Polymerase Chain Reaction; miRNA*,* MicroRNA*

NFKB1 and miRNAs (-342-5p, -5192, and − 15b) are potential biomarkers for diagnosing and monitoring periodontitis and T2DM-associated periodontitis through their association with the inflammatory modulation of both diseases. Their non-invasive nature and strong clinical associations make them promising candidates for personalized management strategies (Fig. 5).

## Supplementary Information

Supplementary Material 1: Supplementary Table S1. workflow for selection of NFKB1/miR-342-5p, -5192, and − 15 b in Type 2 Diabetic Periodontitis., Supplementary Table S2. List of the Primer assays., Supplementary Table S3. Correlation analysis between periodontitis biomarkers and mRNA-miRNAs in serum and GCF., Supplementary Table S4. The post-hoc sensitivity analyses excluding former smokers., Supplementary Table S5. Primary Regression Model to To BMI mitigate confounding effect., Supplementary Table S6. Spearman’s Correlation (ρ) Between Molecular Markers and histopathological Periodontitis Severity Grades.

Supplementary Material 2.

Supplementary Material 3.

## Data Availability

Data presented are available upon reasonable request from the corresponding author.

## Footnotes

1 Nahid MA, Rivera M, Lucas A, Chan EK, Kesavalu L. Polymicrobial infection with periodontal pathogens specifically enhances microRNA miR-146a in ApoE-/- mice during experimental periodontal disease. Infect Immun. 2011 Apr;79(4):1597 − 605. doi: 10.1128/IAI.01062-10. Epub 2011 Jan 24. PMID: 21,263,019; PMCID: PMC3067556.

2 Agnihotri R, Gaur S, Bhat SG. Role of microRNAs in Diabetes-Associated Periodontitis: A Scoping Review. J Int Soc Prev Community Dent. 2024 Jun 27;14(3):180–191. doi: 10.4103/jispcd.jispcd_3_24. PMID: 39,055,291; PMCID: PMC11268527.

3 Venugopal P, Koshy T, Lavu V, Ranga Rao S, Ramasamy S, Hariharan S, Venkatesan V. Differential expression of microRNAs let‐7a, miR‐125b, miR‐100, and miR‐21 and interaction with NF‐kB pathway genes in periodontitis pathogenesis. Journal of cellular physiology. 2018 Aug;233(8):5877-84.
