# Supplementary material for: Identification of NFKB1, miR-342-5p, -5192, and − 15b as diagnostic biomarkers for periodontitis in type 2 diabetes mellitus: a cross-sectional and experimental study
Source: BMC Oral Health. 2026 Feb 23;26:545. doi: 10.1186/s12903-026-07780-2 (PMC13033223; doi:10.1186/s12903-026-07780-2)

**Supplementary Tables**

**Supplementary Table S1 workflow for selection of NFKB1/miR-342-5p, -5192, and -15 b in Type 2 Diabetic Periodontitis**

| **Step** | **Objective / Rationale** | **Database / Tool (Accessed Dec 2024)** | **Input / Search Parameters** | **Analysis Criteria / Algorithms** | **Output / Data Generated** |
| --- | --- | --- | --- | --- | --- |
| **1. Primary miRNA screening** | Identify miRNAs associated with insulin signaling, diabetes, and immune regulation. | **miRNet** (<https://www.mirnet.ca/miRNet/home.xhtml>) | Keywords: “Diabetes Mellitus”, “Type 2 Diabetes” | Network-based integration of miRNA–gene–disease interactions; top miRNAs selected based on degree centrality and frequency of association. | Candidate miRNAs: *hsa-miR-342-5p*, *hsa-miR-15b* |
| **2. Cross-validation of disease association** | Confirm reproducibility of candidate miRNAs across independent datasets. | **HMDD v4.0** (<http://www.cuilab.cn/hmdd>) and **miRTarBase** (<https://mirtarbase.cuhk.edu.cn/~miRTarBase/miRTarBase_2025/php/index.php>) | Disease terms: “Type 2 Diabetes”, “Insulin Resistance”. | Inclusion of miRNAs with *experimentally validated* interactions from ≥2 datasets. | Cross-validated list of miRNAs consistently linked to diabetes. |
| **3. Novel candidate inclusion** | Integrate additional miRNAs of mechanistic importance to T2DM (validated by previous work). | Literature & prior validation | – | Inclusion based on a previously observed correlation between *miR-5192* and insulin resistance / inflammatory activation. | Extended biomarker panel (*miR-342-5p*, *miR-15b*, *miR-5192*). |
| **4. Functional pathway enrichment** | Elucidate biological pathways linked to the selected miRNAs. | **miRPathDB** (<https://mpd.bioinf.uni-sb.de/overview.html>) | Input: selected miRNAs (3 total). | Significance threshold *P* < 0.05; enrichment score > 1.5; KEGG & Reactome pathway mapping; Gene Ontology (GO) confirmation. | Enriched pathways in “insulin signaling,” “immune response regulation,” and “NF-κB activation.” |
| **5. Target prediction of miRNA–NFKB1 interaction** | Identify direct binding and regulatory relationships between candidate miRNAs and NFKB1 gene. | **miRWalk** (<http://mirwalk.umm.uni-heidelberg.de/>) | Target gene: *NFKB1*. | Inclusion threshold: prediction score ≥ 0.9; validated by ≥3 prediction algorithms (miRanda, TargetScan, RNAhybrid). | High-confidence *miRNA–NFKB1* interaction map (Fig. S4). |
| **6. Tissue-specific expression validation** | Confirm biological plausibility and translational relevance in oral and systemic tissues. | **miRNA Tissue Atlas** (<https://ccb-compute2.cs.uni-saarland.de/mirnatissueatlas_2025/>) | Query: *miR-342-5p*, *miR-15b*, *miR-5192*. | Comparative expression across blood, saliva, gingival, and pancreatic tissues. | Verified expression of all selected miRNAs in oral and circulatory compartments. |
| **7. Literature synthesis** | Integrate computational findings with published evidence. | PubMed & cited studies [1–4]. | Keywords: “miR-342-5p”, “miR-15b”, “miR-5192”, “diabetes”, “periodontitis”. | Inclusion of experimentally validated reports supporting their inflammatory and metabolic functions. | Consolidated biological rationale for final biomarker selection. |

**Supplementary Table S2.** List of the Primer assays.

| miRNA | Catalog number | GeneGlobe ID |
| --- | --- | --- |
| hsa-miR-15b | Cat. No.: 1027416 | GS406949 |
| rno-miR-15b-5p | Cat. No.: 219600 | MSY0000784 |
| [hsa-miR-342-5p](https://geneglobe.qiagen.com/eg/product-groups/mircury-lna-mirna-pcr-assays/YP00204516) | Cat. No.: 339306 | YP00204516 |
| rno-miR-342-5p | Cat. No.: 219600 | MSY0004652 |
| has-miR-5192 | Cat. No.: 219600 | MSY0021123 |
| RNU6-2 | Cat. No.: MS00033740 | SBH0165830 |

MiR:Microribonucleic acid, RNU6-2: RNA, U6B Small Nuclear, Cat. No: Catalog number.

**Supplementary Table S3. Correlation analysis between periodontitis biomarkers and mRNA-miRNAs in serum and GCF.**

|  | | | **CAL(mm)** | **BOP %** | **Mean PLI** | **PPD (mm)** | **NFKB1 (G)** | **NFKB1 (S)** | **Hsa-miR-15b (G)** | **Hsa-miR-15b (S)** | **Hsa-miR-5192 (G)** | **Hsa-miR-15b (S)** | **Hsa-miR-342-5p (G)** | **Hsa-miR-342-5p (S)** |
| --- | --- | --- | --- | --- | --- | --- | --- | --- | --- | --- | --- | --- | --- | --- |
| Spearman's rho | **CAL (mm)** | **Correlation Coefficient** | 1.000 | .748^**^ | .841^**^ | .878^**^ | .865^**^ | .839^**^ | .859^**^ | .863^**^ | .852^**^ | .870^**^ | .762^**^ | .809^**^ |
|  |  | **Sig. (2-tailed)** | . | .000 | .000 | .000 | .000 | .000 | .000 | .000 | .000 | .000 | .000 | .000 |
|  |  | **N** | 140 | 140 | 140 | 140 | 32 | 140 | 32 | 140 | 32 | 140 | 32 | 140 |
|  | **Bleeding on probing %** | **Correlation Coefficient** | .748^**^ | 1.000 | .737^**^ | .740^**^ | .576^**^ | .704^**^ | .545^**^ | .680^**^ | .629^**^ | .709^**^ | .633^**^ | .695^**^ |
|  |  | **Sig. (2-tailed)** | .000 | . | .000 | .000 | .001 | .000 | .001 | .000 | .000 | .000 | .000 | .000 |
|  |  | **N** | 140 | 140 | 140 | 140 | 32 | 140 | 32 | 140 | 32 | 140 | 32 | 140 |
|  | **Mean PLI** | **Correlation Coefficient** | .841^**^ | .737^**^ | 1.000 | .795^**^ | .651^**^ | .788^**^ | .746^**^ | .816^**^ | .657^**^ | .772^**^ | .607^**^ | .751^**^ |
|  |  | **Sig. (2-tailed)** | .000 | .000 | . | .000 | .000 | .000 | .000 | .000 | .000 | .000 | .000 | .000 |
|  |  | **N** | 140 | 140 | 140 | 140 | 32 | 140 | 32 | 140 | 32 | 140 | 32 | 140 |
|  | **PPD (mm)** | **Correlation Coefficient** | .878^**^ | .740^**^ | .795^**^ | 1.000 | .902^**^ | .815^**^ | .885^**^ | .860^**^ | .866^**^ | .835^**^ | .769^**^ | .780^**^ |
|  |  | **Sig. (2-tailed)** | .000 | .000 | .000 | . | .000 | .000 | .000 | .000 | .000 | .000 | .000 | .000 |
|  |  | **N** | 140 | 140 | 140 | 140 | 32 | 140 | 32 | 140 | 32 | 140 | 32 | 140 |
|  | **NFKB1 (G)** | **Correlation Coefficient** | .865^**^ | .576^**^ | .651^**^ | .902^**^ | 1.000 | .923^**^ | .879^**^ | .872^**^ | .892^**^ | .880^**^ | .851^**^ | .882^**^ |
|  |  | **Sig. (2-tailed)** | .000 | .001 | .000 | .000 | . | .000 | .000 | .000 | .000 | .000 | .000 | .000 |
|  |  | **N** | 32 | 32 | 32 | 32 | 32 | 32 | 32 | 32 | 32 | 32 | 32 | 32 |
|  | **NFKB1 (S)** | **Correlation Coefficient** | .839^**^ | .704^**^ | .788^**^ | .815^**^ | .923^**^ | 1.000 | .847^**^ | .790^**^ | .890^**^ | .786^**^ | .837^**^ | .775^**^ |
|  |  | **Sig. (2-tailed)** | .000 | .000 | .000 | .000 | .000 | . | .000 | .000 | .000 | .000 | .000 | .000 |
|  |  | **N** | 140 | 140 | 140 | 140 | 32 | 140 | 32 | 140 | 32 | 140 | 32 | 140 |
|  | **Hsa-miR-15b (G)** | **Correlation Coefficient** | .859^**^ | .545^**^ | .746^**^ | .885^**^ | .879^**^ | .847^**^ | 1.000 | .933^**^ | .862^**^ | .843^**^ | .793^**^ | .818^**^ |
|  |  | **Sig. (2-tailed)** | .000 | .001 | .000 | .000 | .000 | .000 | . | .000 | .000 | .000 | .000 | .000 |
|  |  | **N** | 32 | 32 | 32 | 32 | 32 | 32 | 32 | 32 | 32 | 32 | 32 | 32 |
|  | **Hsa-miR-15b (S)** | **Correlation Coefficient** | .863^**^ | .680^**^ | .816^**^ | .860^**^ | .872^**^ | .790^**^ | .933^**^ | 1.000 | .815^**^ | .807^**^ | .747^**^ | .779^**^ |
|  |  | **Sig. (2-tailed)** | .000 | .000 | .000 | .000 | .000 | .000 | .000 | . | .000 | .000 | .000 | .000 |
|  |  | **N** | 140 | 140 | 140 | 140 | 32 | 140 | 32 | 140 | 32 | 140 | 32 | 140 |
|  | **Hsa-miR-5192 (G)** | **Correlation Coefficient** | .852^**^ | .629^**^ | .657^**^ | .866^**^ | .892^**^ | .890^**^ | .862^**^ | .815^**^ | 1.000 | .949^**^ | .855^**^ | .846^**^ |
|  |  | **Sig. (2-tailed)** | .000 | .000 | .000 | .000 | .000 | .000 | .000 | .000 | . | .000 | .000 | .000 |
|  |  | **N** | 32 | 32 | 32 | 32 | 32 | 32 | 32 | 32 | 32 | 32 | 32 | 32 |
|  | **Hsa-miR-5192 (S)** | **Correlation Coefficient** | .870^**^ | .709^**^ | .772^**^ | .835^**^ | .880^**^ | .786^**^ | .843^**^ | .807^**^ | .949^**^ | 1.000 | .845^**^ | .781^**^ |
|  |  | **Sig. (2-tailed)** | .000 | .000 | .000 | .000 | .000 | .000 | .000 | .000 | .000 | . | .000 | .000 |
|  |  | **N** | 140 | 140 | 140 | 140 | 32 | 140 | 32 | 140 | 32 | 140 | 32 | 140 |
|  | **Hsa-miR-342-5p (G)** | **Correlation Coefficient** | .762^**^ | .633^**^ | .607^**^ | .769^**^ | .851^**^ | .837^**^ | .793^**^ | .747^**^ | .855^**^ | .845^**^ | 1.000 | .973^**^ |
|  |  | **Sig. (2-tailed)** | .000 | .000 | .000 | .000 | .000 | .000 | .000 | .000 | .000 | .000 | . | .000 |
|  |  | **N** | 32 | 32 | 32 | 32 | 32 | 32 | 32 | 32 | 32 | 32 | 32 | 32 |
|  | **Hsa-miR-342-5p (S)** | **Correlation Coefficient** | .809^**^ | .695^**^ | .751^**^ | .780^**^ | .882^**^ | .775^**^ | .818^**^ | .779^**^ | .846^**^ | .781^**^ | .973^**^ | 1.000 |
|  |  | **Sig. (2-tailed)** | .000 | .000 | .000 | .000 | .000 | .000 | .000 | .000 | .000 | .000 | .000 | . |
|  |  | **N** | 140 | 140 | 140 | 140 | 32 | 140 | 32 | 140 | 32 | 140 | 32 | 140 |
| **. Correlation is significant at the 0.01 level (2-tailed). | | | | | | | | | | | | | | |

Abbreviations: G, gingival crevicular fluid; S: Serum. **ρ (Spearman’s rho)**: Correlation coefficient (−1 to +1).. Significance levels: p < 0.05*, p < 0.01*, **p < 0.001.

**Supplementary Table S4:The post-hoc sensitivity analyses excluding former smokers**

| **Biomarker** | **Group Comparison** | **Adjusted p-value** | **Effect Size (95% CI)** | **Δ Effect Size** | **Clinical Correlation (r [95% CI])** |
| --- | --- | --- | --- | --- | --- |
| **NFKB1** | T2DM+CP vs. CP (non-diabetic) | 0.018 | Cohen’s *d* = 1.18 (0.82–1.54) | -0.24 vs. full cohort | CAL: 0.72 (0.65–0.78) HbA1c: 0.65 (0.57–0.71) |
| **miR-342-5p** | T2DM+CP vs. CP (non-diabetic) | 0.023 | Hedges’ *g* = 1.32 (0.95–1.69) | -12% | CAL: 0.70 (0.63–0.76) HbA1c: 0.63 (0.55–0.69) |
| **miR-5192** | T2DM+CP vs. CP (non-diabetic) | 0.031 | Hedges’ *g* = 1.21 (0.84–1.58) | -9% | CAL: 0.68 (0.60–0.74) HbA1c: 0.61 (0.53–0.67) |
| **miR-15b** | T2DM+CP vs. CP (non-diabetic) | 0.019 | Hedges’ *g* = 1.45 (1.07–1.83) | -15% | CAL: 0.71 (0.64–0.77) HbA1c: 0.64 (0.56–0.70) |

**Sample Size After Exclusion**:T2DM+CP: n = 84 (originally n = 84, 0 former smokers), Non-diabetic CP: n = 66 (originally n = 90, 24 former smokers excluded), Controls: n = 54 (originally n = 90, 36 former smokers excluded). **Statistical Adjustments**: Covariates included age, sex, and BMI.**Effect Size Interpretation**:Cohen’s *d*: 0.8 = large effect Hedges’ *g*: 0.5 = medium, 0.8 = large

#### **Supplementary Table S5:Primary Regression Model** to To BMI mitigate confounding effect

| **Variable** | **Outcome: Fasting Glucose (β, 95% CI)** | **Outcome: HbA1c (β, 95% CI)** | **p-value** |
| --- | --- | --- | --- |
| **Disease Status** | 38.2 (34.1, 42.3) | 1.6 (1.3, 1.9) | <0.001* |
| **BMI (per 1 kg/m²)** | 2.4 (1.9, 2.9) | 0.08 (0.05, 0.11) | <0.001* |
| **Interaction (Disease × BMI)** | 1.2 (0.7, 1.7) | 0.04 (0.02, 0.06) | 0.01* |
| **Adjusted R²** | 0.78 | 0.65 | — |

Adjusted for age, sex, smoking status, and baseline comorbidities.Interaction term (Disease × BMI) tests whether BMI effects differ between disease and control groups.

### **Supplementary Table S6 :Spearman’s Correlation (ρ) Between Molecular Markers and histopathological Periodontitis Severity Grades**

| **Molecular Marker** | **Non-Diabetic + Ligation Group (ρ, p-value)** | **Diabetic + Ligation Group (ρ, p-value)** |
| --- | --- | --- |
| NFKB1 | Mild: 0.72*** (<0.001) | Mild: 0.85*** (<0.001) |
|  | Moderate: 0.83*** (<0.001) | Moderate: 0.89*** (<0.001) |
|  | Severe: 0.92*** (<0.001) | Severe: 0.95*** (<0.001) |
| rno-miR-15b | Mild: 0.40* (0.032) | Mild: 0.45* (0.018) |
|  | Moderate: 0.52** (0.006) | Moderate: 0.60** (0.002) |
|  | Severe: 0.63*** (<0.001) | Severe: 0.75*** (<0.001) |
| rno-miR-342-5p | Mild: 0.30 (0.105) | Mild: 0.58* (0.025) |
|  | Moderate: 0.45* (0.022) | Moderate: 0.70** (0.003) |
|  | Severe: 0.55** (0.005) | Severe: 0.78*** (<0.001) |

- **ρ (Spearman’s rho)**: Correlation coefficient (−1 to +1).. Significance levels: p < 0.05*, p < 0.01*, **p < 0.001.**Severity Grading**: Mild: Limited inflammation (Score 1–3).Moderate: Localized bone resorption (Score 4–6). Severe: Widespread bone loss (Score 7–9).

**Supplementary Figures**

**Figure S1.** Identification of diabetes-associated miRNAs using the miRNet database.


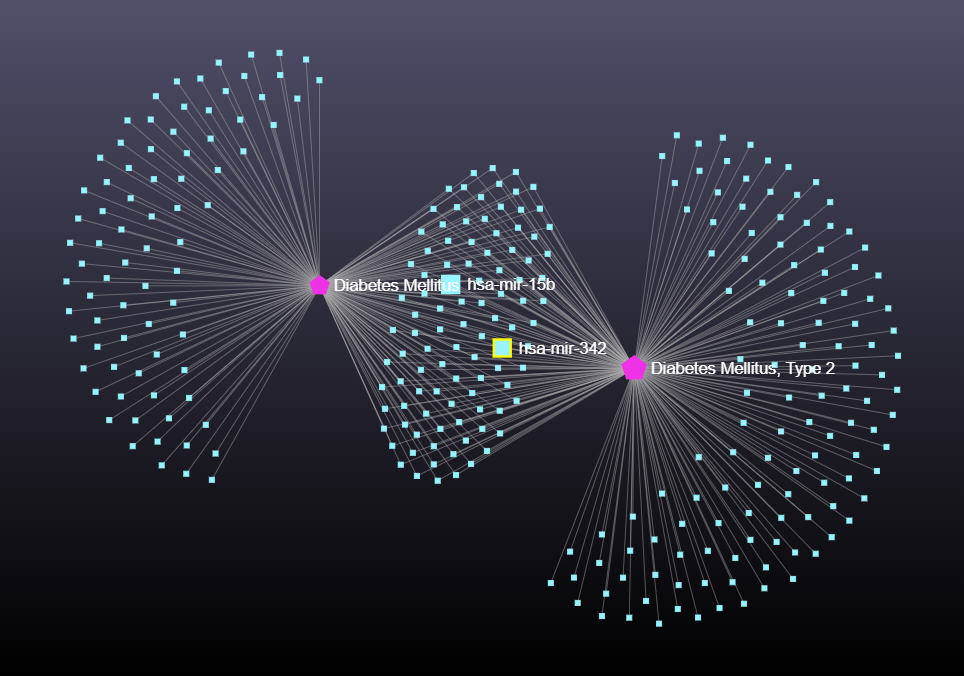


**Figure S2*.*** Validation of candidate miRNAs using HMDD v4.0 and miRTarBase databases.

**
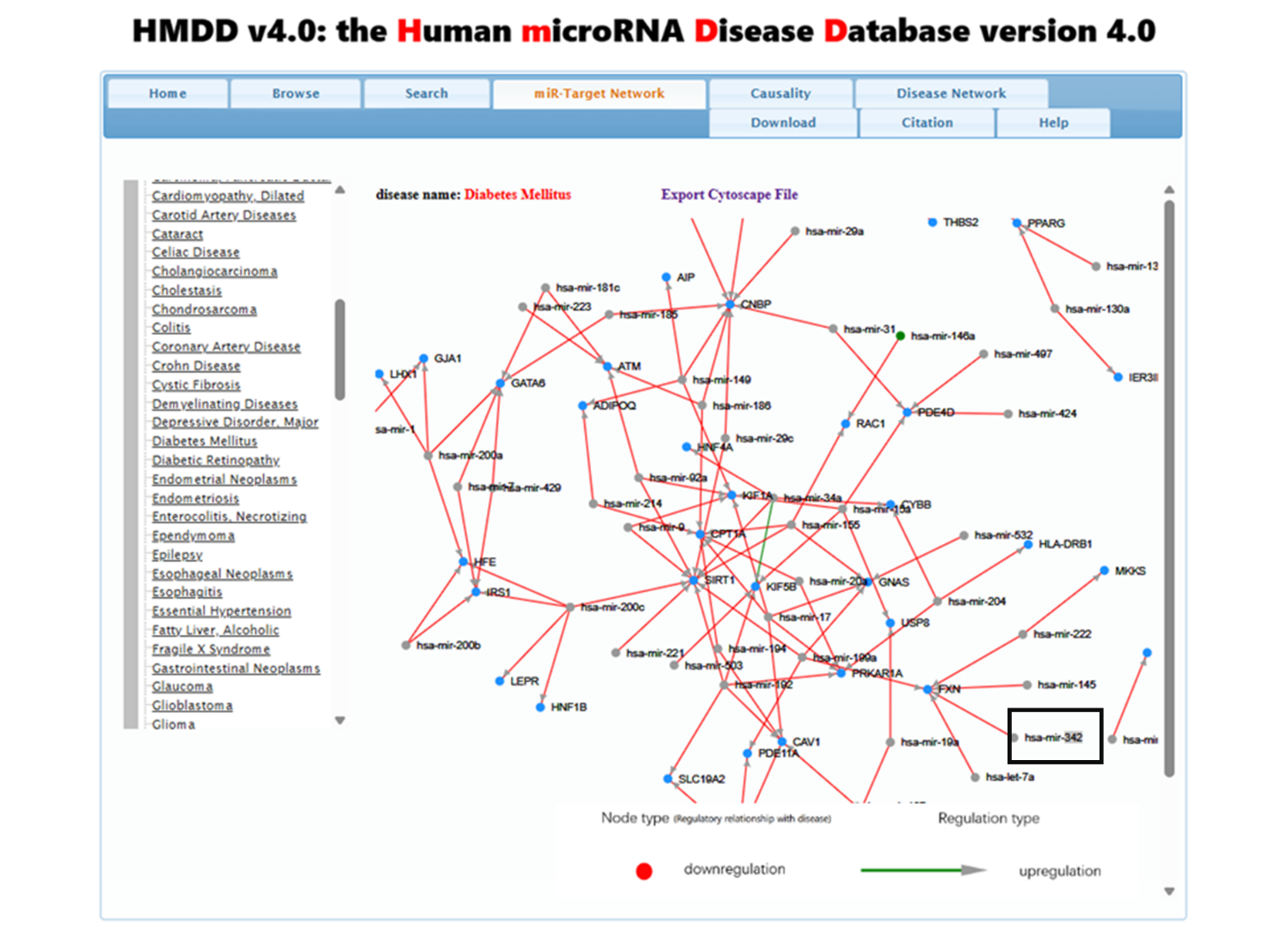
**

**
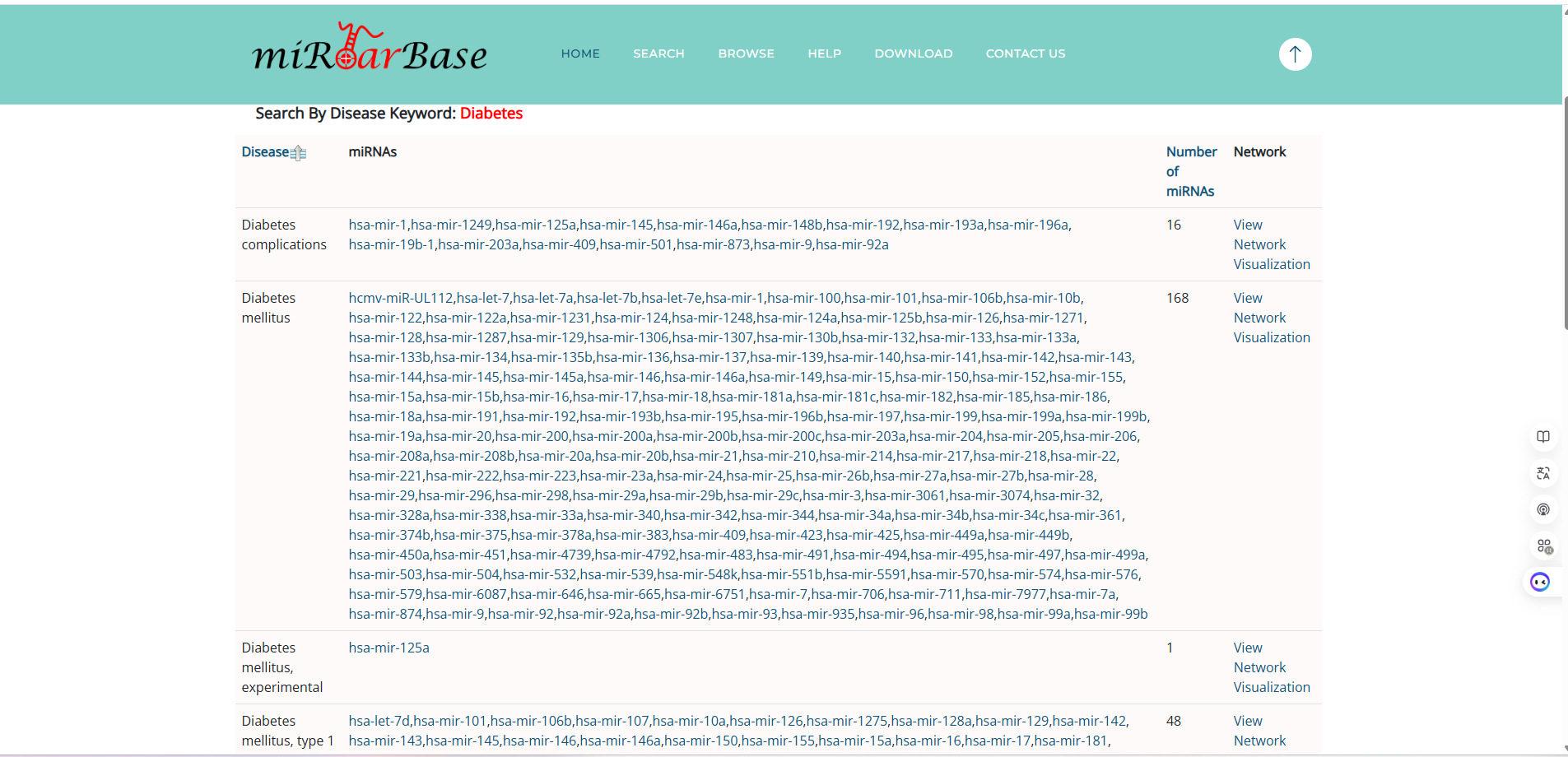
**

**Figure S3. Enrichment analysis of the 3 miRNAs by miRPathDB.**

**
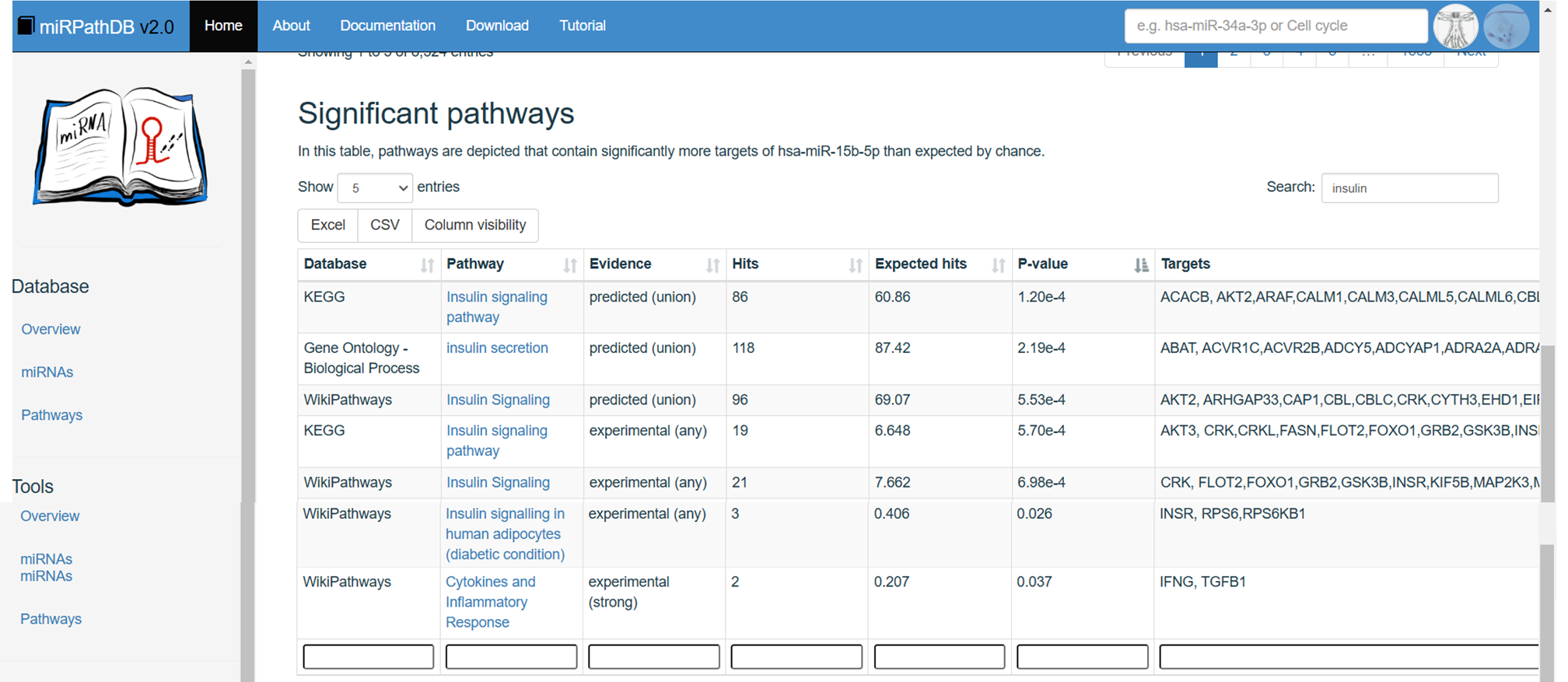
**

**
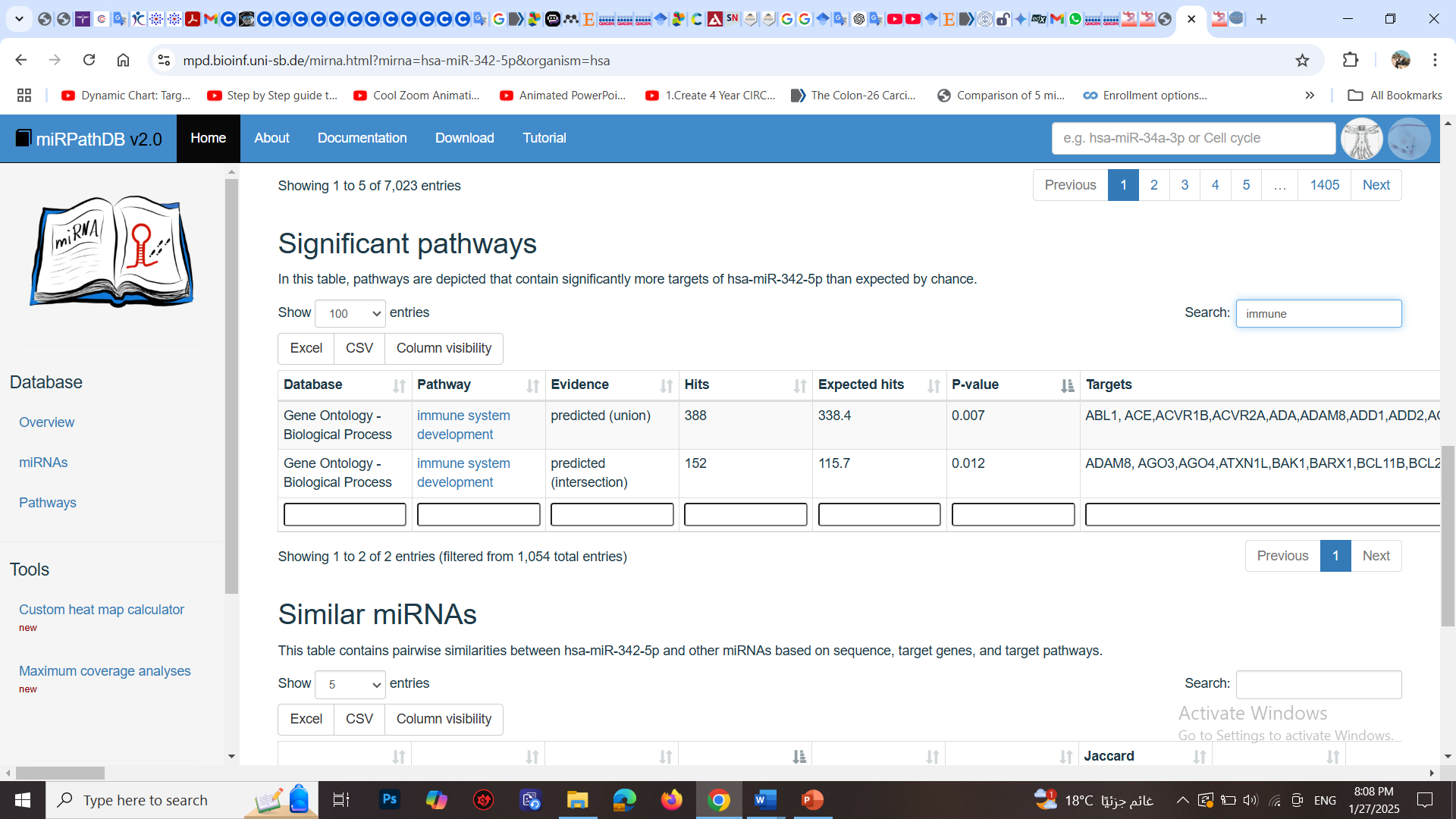
**

**
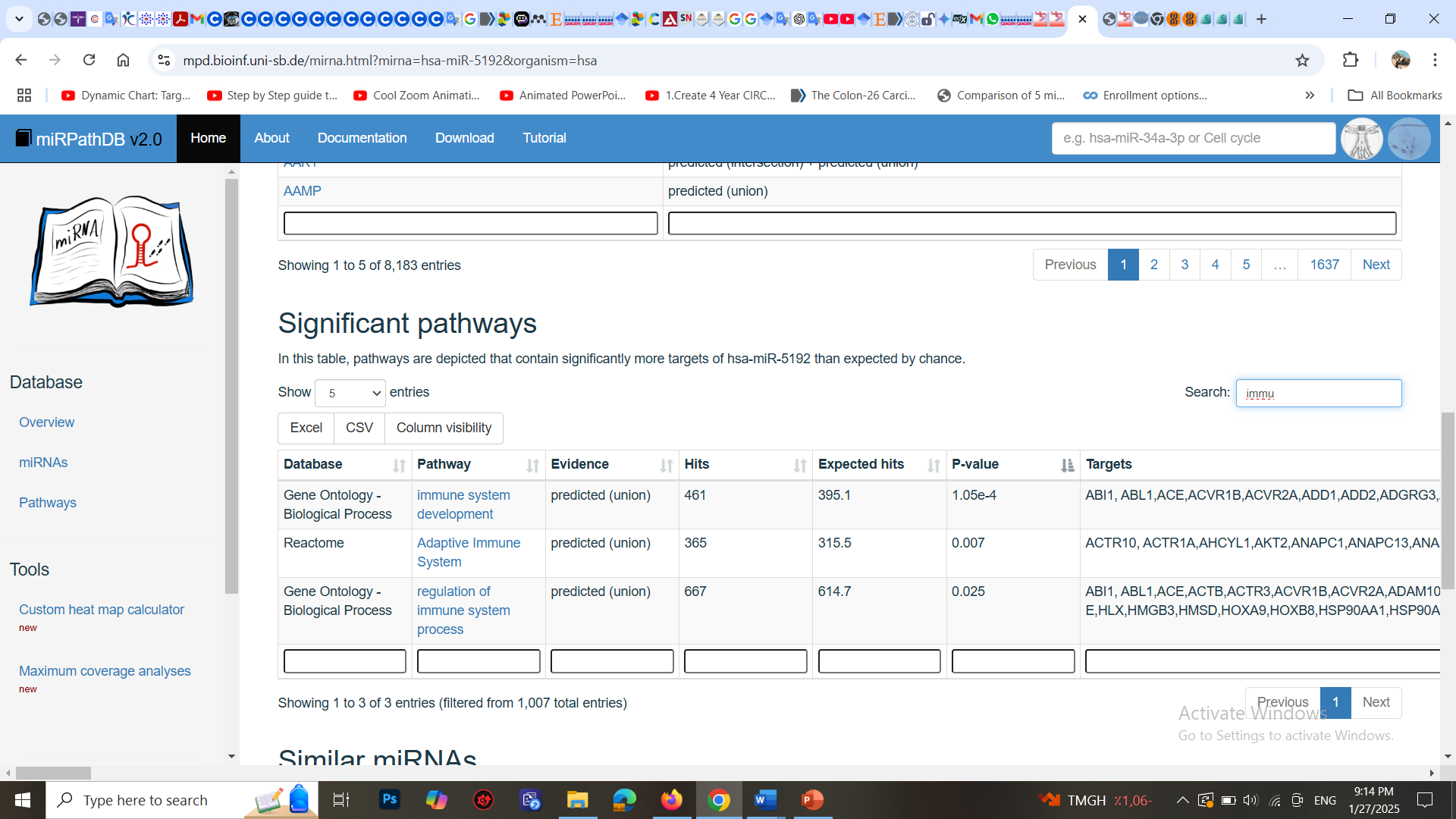
**

**Figure S4.** Predicted miRNAs interacting with NF-KB1, identified using the miRWalk database.


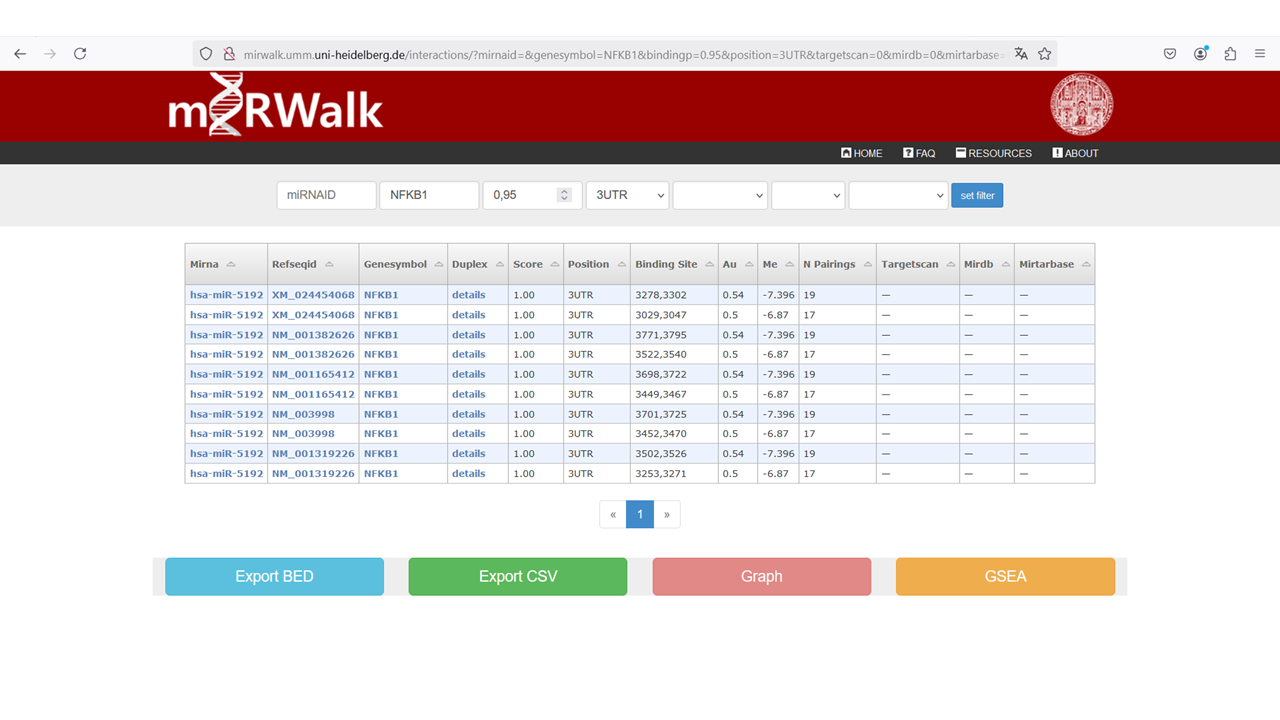

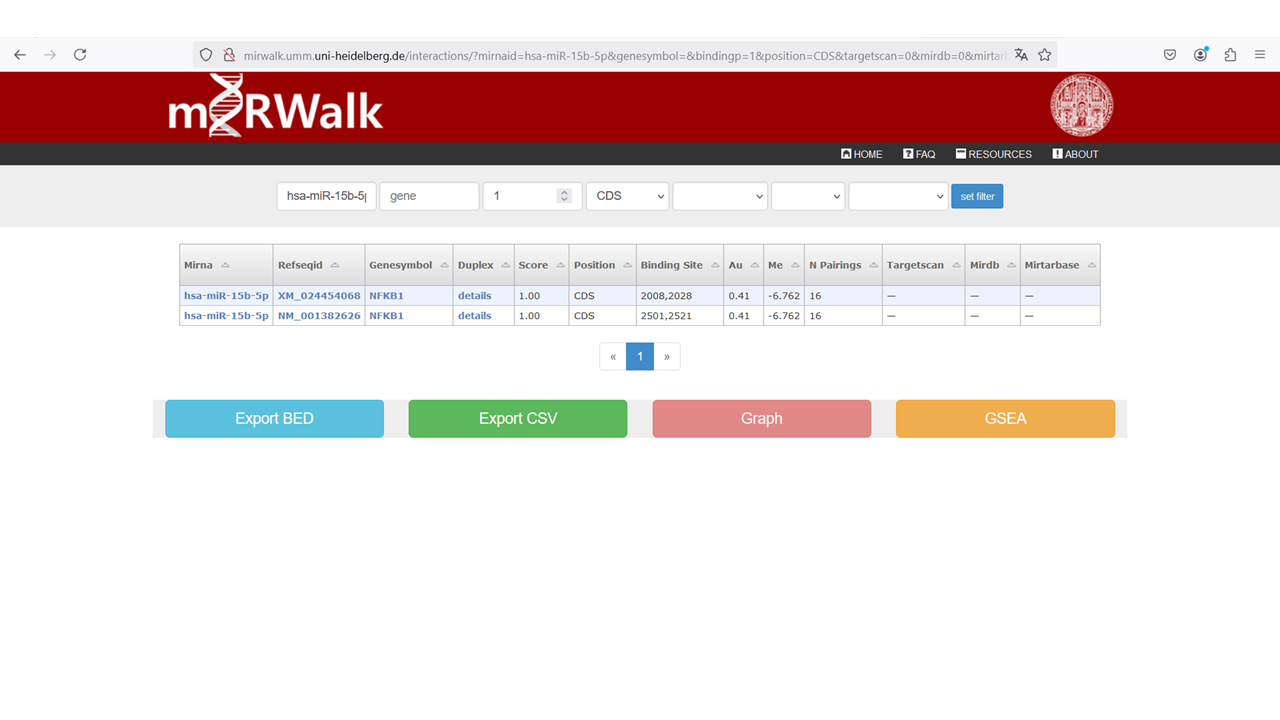


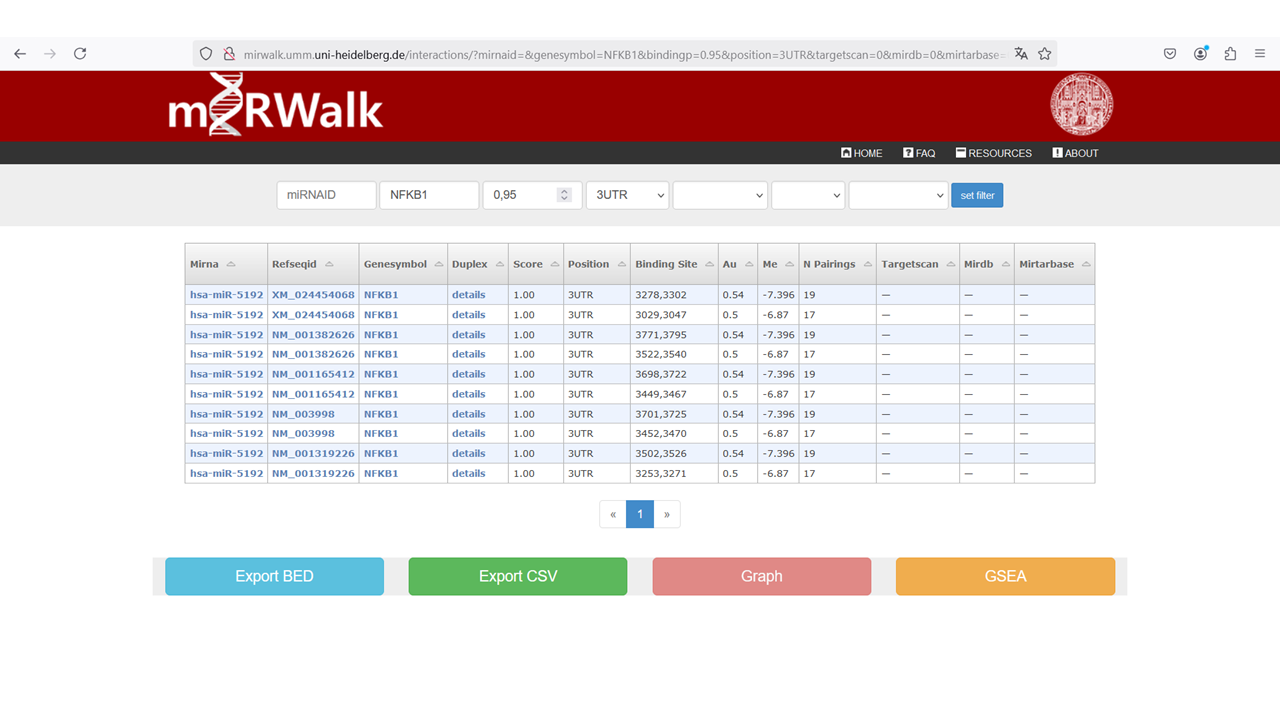


**Figure S5.** Expression of candidate miRNAs in blood, saliva, and the oral cavity, as confirmed by the miRNA Tissue Atlas database.

1. hsa-miR-5192


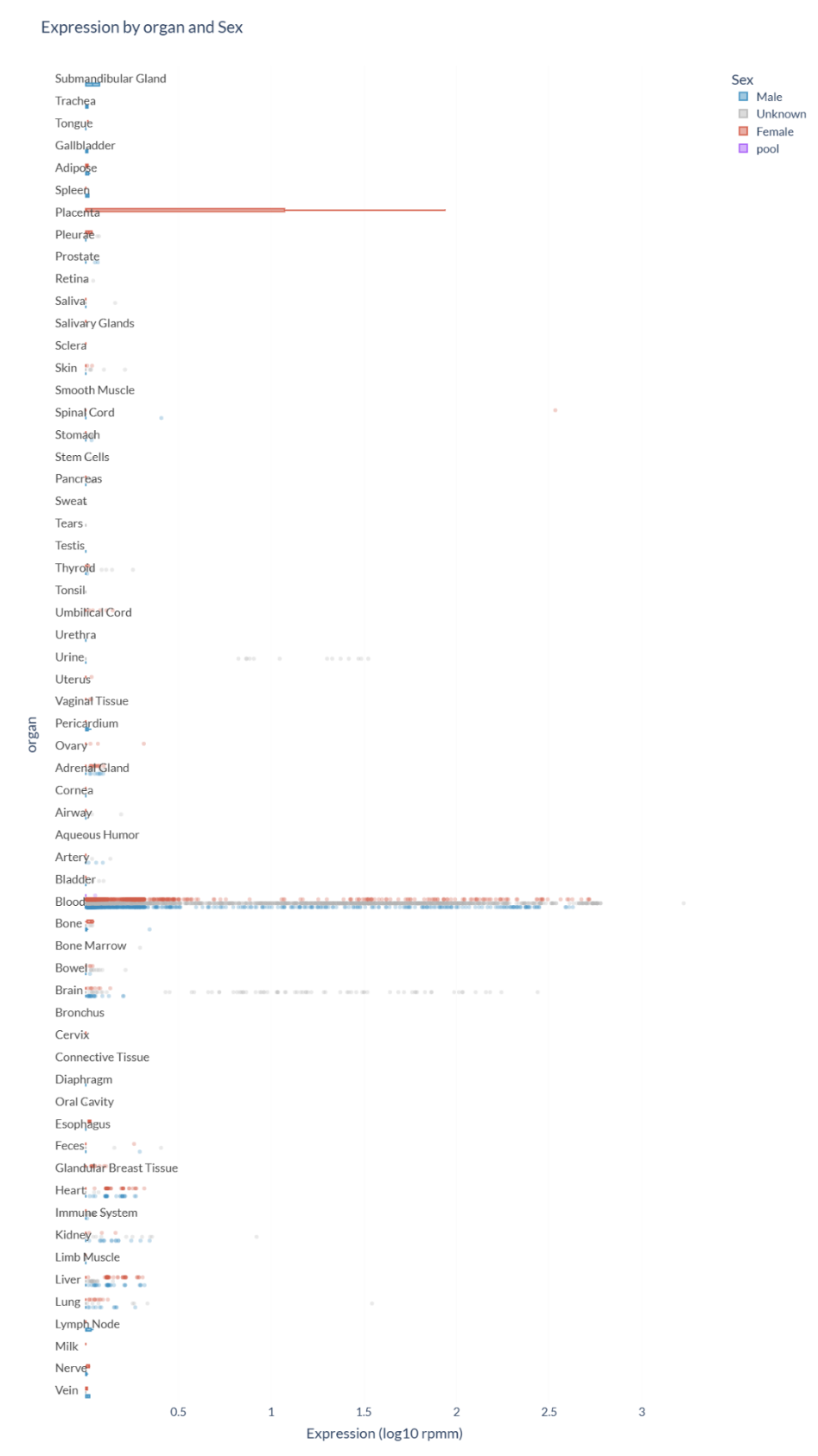


1. hsa-miR-15b


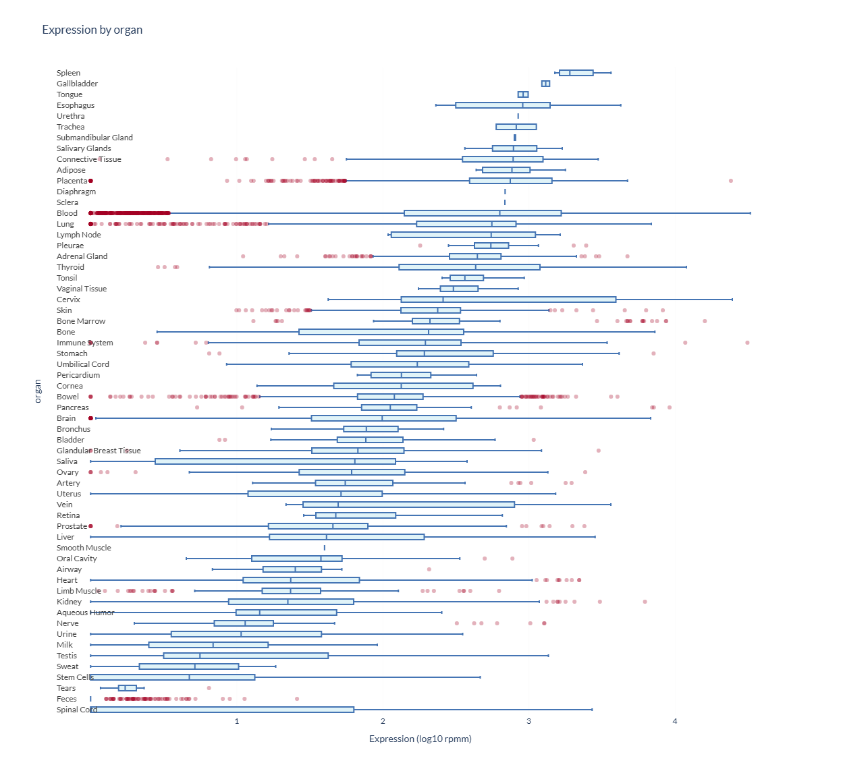


hsa-miR-342-5p


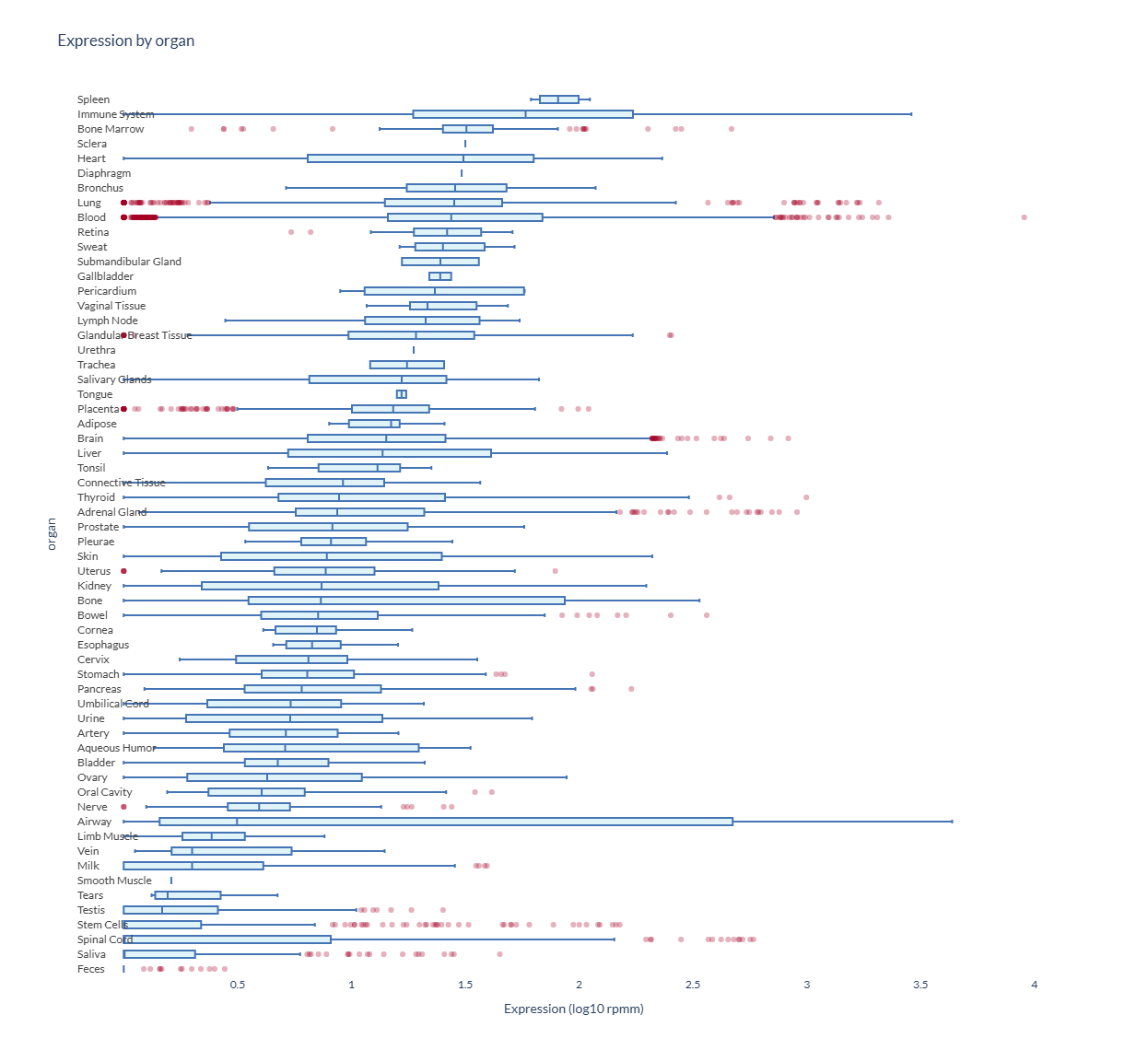


**Figure S6.** **Differential Expression of Serum and GCF mRNA-miRNAs.(A) the relative expression of NFKB (G), (B) the relative expression of NFKB (S), (C) the relative expression of** hsa-miR-15b (G), (D) **the relative expression of** hsa-miR-15b (S), (E) **the relative expression of** hsa-miR-5192 (G), **the relative expression of** hsa-miR-5192 (S), (F) **the relative expression of** hsa-miR-342(G), and (H) **the relative expression of** hsa-miR-342 (S).


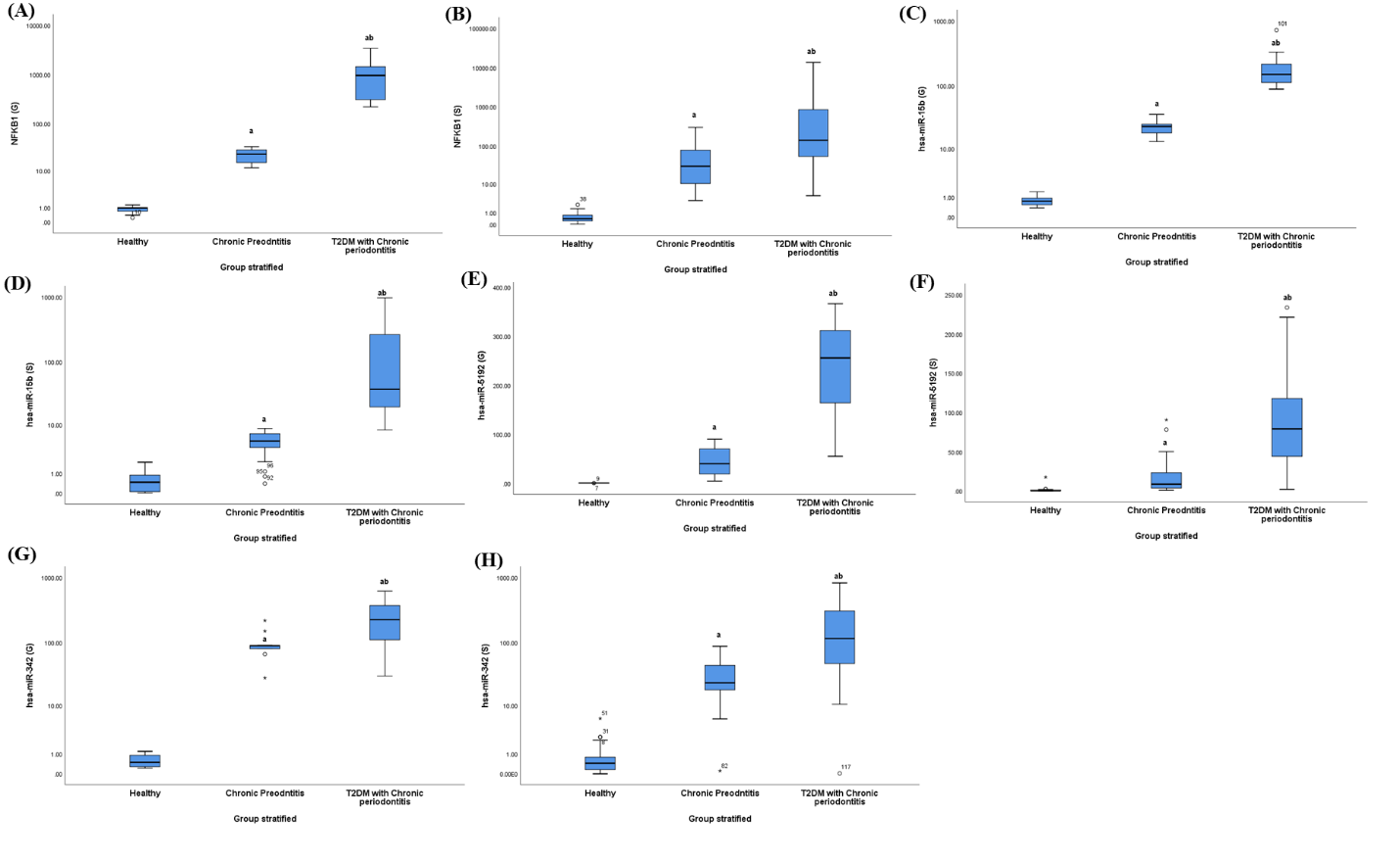


**Figure S7.** ROC curves representing the investigated serum and GCF NFKB1-miRNAs between the healthy and both patients’ groups. **(A) NFKB (G), (B) NFKB (S), (C)** hsa-miR-15b (G), (D) hsa-miR-15b (S), (E) hsa-miR-5192 (G), hsa-miR-5192 (S), (F) hsa-miR-342(G), and **(H)** hsa-miR-342 (S).


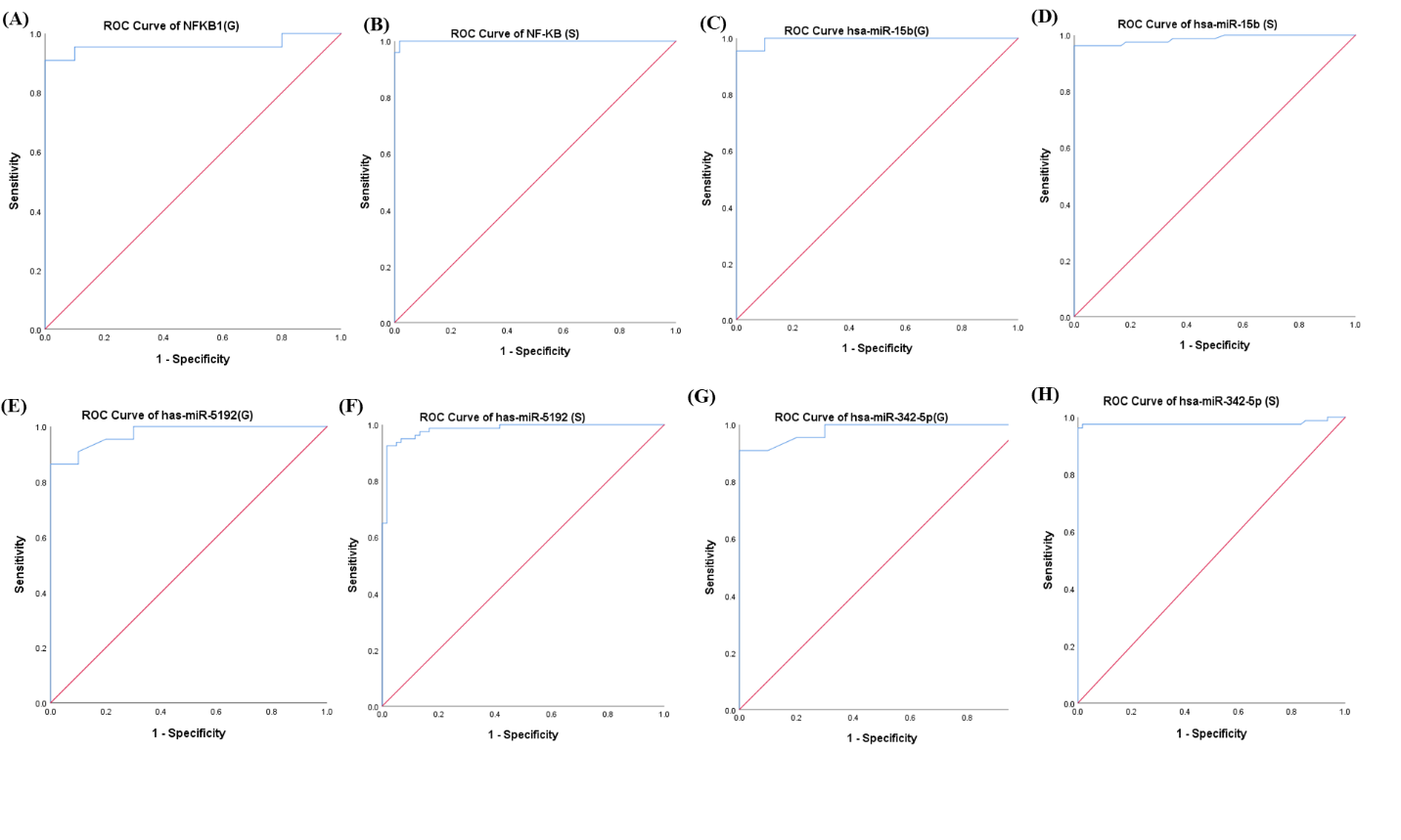


**Figure S8.** ROC curves representing the investigated serum and GCF NFKB1-miRNAs between Chronic periodontitis and T2DM with Chronic periodontitis. **(A) NFKB (G), (B) NFKB (S), (C)** hsa-miR-15b (G), (D) hsa-miR-15b (S), (E) hsa-miR-5192 (G), hsa-miR-5192 (S), (F) hsa-miR-342(G), and **(H)** hsa-miR-342 (S).


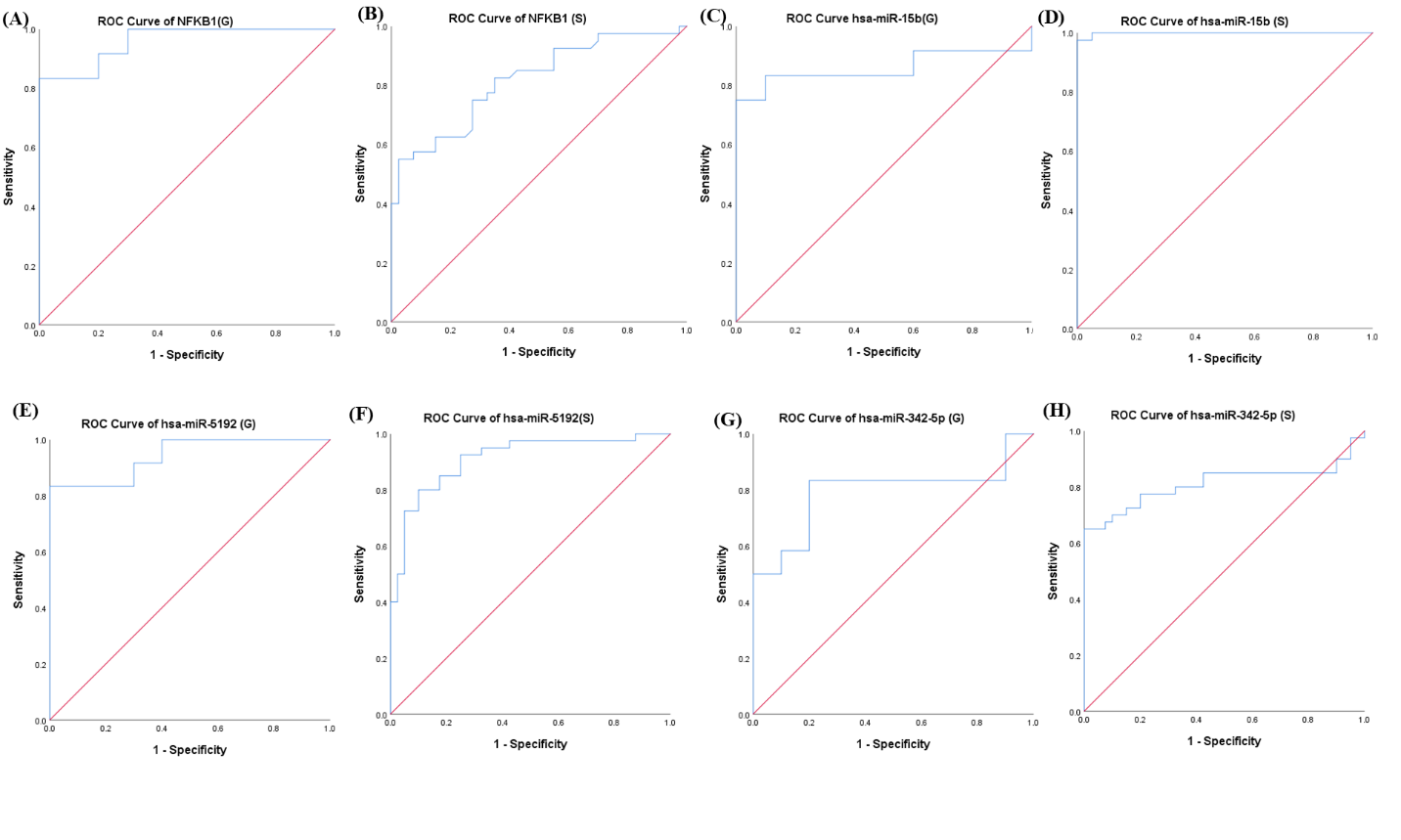


**Figure S9.** ROC curves representing the investigated serum and GCF NFKB1-miRNAs between Healthy control and Chronic periodontitis. **(A) NFKB (G), (B) NFKB (S), (C)** hsa-miR-15b (G), (D) hsa-miR-15b (S), (E) hsa-miR-5192 (G), hsa-miR-5192 (S), (F) hsa-miR-342(G), and **(H)** hsa-miR-342 (S).


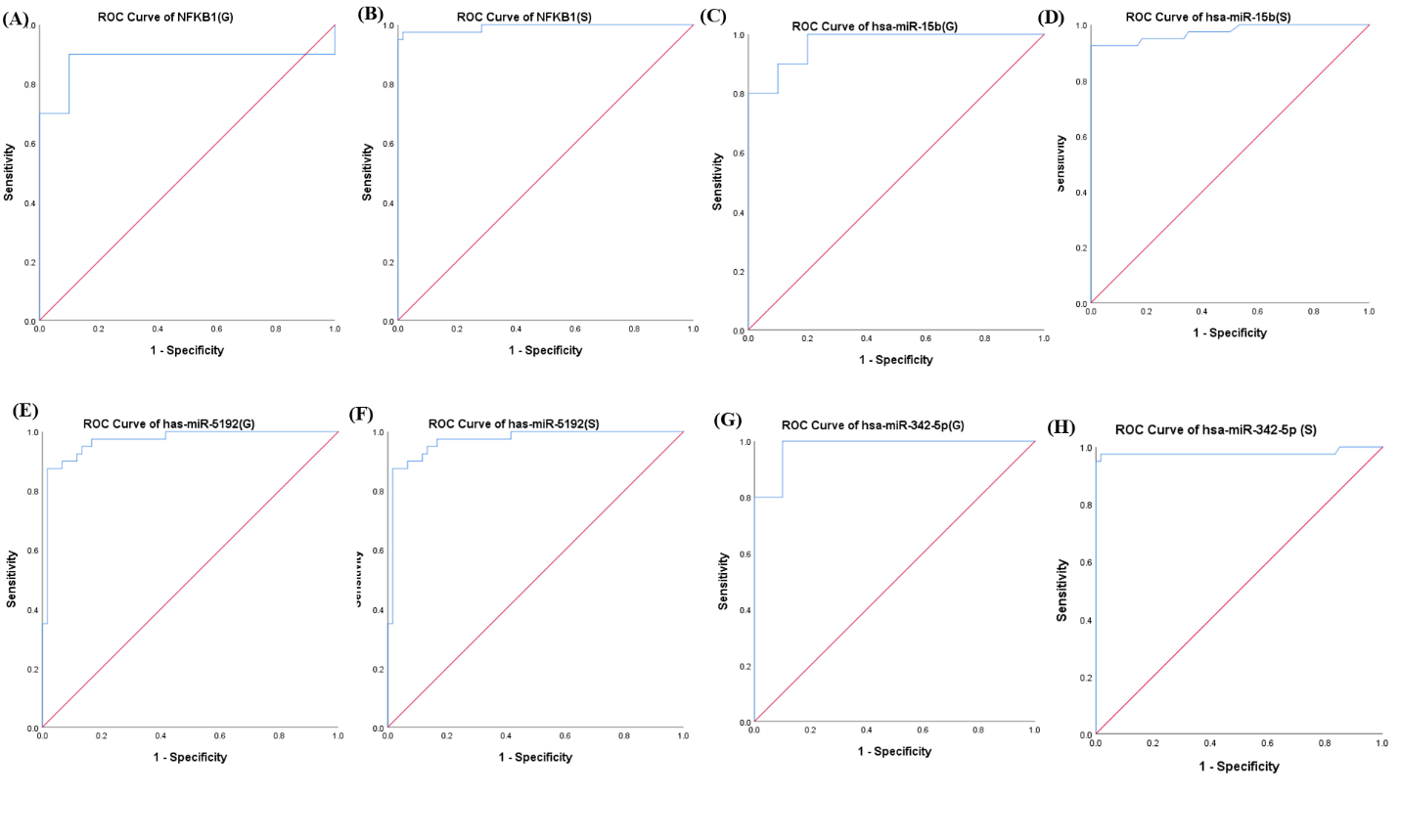

Supplement: Supplementary file 1 — Supplementary Material 1: Supplementary Table S1. workflow for selection of NFKB1/miR-342-5p, -5192, and − 15 b in Type 2 Diabetic Periodontitis., Supplementary Table S2. List of the Primer assays., Supplementary Table S3. Correlation analysis between periodontitis biomarkers and mRNA-miRNAs in serum and GCF., Supplementary Table S4. The post-hoc sensitivity analyses excluding former smokers., Supplementary Table S5. Primary Regression Model to To BMI mitigate confounding effect., Supplementary Table S6. Spearman’s Correlation (ρ) Between Molecular Markers and histopathological Periodontitis Severity Grades. [file 12903_2026_7780_MOESM1_ESM.docx]
