# Supplementary material for: Identification of NFKB1, miR-342-5p, -5192, and − 15b as diagnostic biomarkers for periodontitis in type 2 diabetes mellitus: a cross-sectional and experimental study
Source: BMC Oral Health. 2026 Feb 23;26:545. doi: 10.1186/s12903-026-07780-2 (PMC13033223; doi:10.1186/s12903-026-07780-2)
Supplement: Supplementary file 3 — Supplementary Material 3. [file 12903_2026_7780_MOESM3_ESM.docx]

| **Item** | **Recommendation** | **Section / Line Number or Explanation** |
| --- | --- | --- |
| **1. Study design** | a. Groups being compared, including controls. b. Experimental unit. | **Methods: Study Design (lines 169–254)** —   - Human study: 3 groups (Healthy control n=60, Chronic periodontitis n=40, T2DM+periodontitis n=40). - Animal study: 3 groups (Healthy control Wistar rats n=12, Chronic periodontitis n=12, T2DM+periodontitis GK rats n=12). Experimental unit = individual participant or individual rat. |
| **2. Sample size** | a. Exact n per group and total. b. Explain sample size determination or calculation. | **Methods: Study Design (lines 171–174, 211–227)** — Human: n=140 total (60+40+40). Animal: n=36 total (12 per group). |
| **3. Inclusion/exclusion criteria** | a. Describe inclusion/exclusion criteria. b. Report excluded data/animals. c. Report n per analysis. | **Methods: Human Model (lines 171–181)** — Inclusion: adults >19 y, BMI < 25 kg/m², ≥20 teeth, no systemic illness except T2DM. Exclusion: recent therapy, pregnancy, implants, antibiotics/steroids <6 mo. **No exclusions reported** after enrollment. Exact n per analysis indicated in Tables 1–7. |
| **4. Randomisation** | a. Was randomisation used? b. Describe control for confounders. | **Methods: Animal Model (lines 210–254)** — Random allocation of rats to 3 experimental groups. No randomisation used in human sample (consecutive enrollment). |
| **5. Blinding** | Describe who was aware of group allocation. | **Not explicitly blinded**, but clinical assessors were independent |
| **6. Outcome measures** | a. Define all outcomes. b. Specify primary outcome. | **Methods: Quantitative PCR and ELISA (lines 193–209)** — Primary outcomes: NFKB1 and miRNA (miR-342-5p, miR-5192, miR-15b) levels in serum, GCF, and gingival tissues. Secondary: clinical periodontal indices (CAL, PPD, BOP, PLI) and metabolic markers. |
| **7. Statistical methods** | a. Statistical methods for each analysis. b. Check of assumptions. | **Methods: Statistical Analysis (lines 255–263)** — Software: SPSS v25. Normality checked; ANOVA, t-tests, Kruskal-Wallis, chi-square, Tukey’s and Mann-Whitney U tests, Spearman correlations, ROC curves. Significance p<0.05. |
| **8. Experimental animals** | a. Species, strain, sex, age, weight. b. Provenance, health, genotype, previous procedures. | **Methods: Animal Model (lines 210–227)** — Male Goto-Kakizaki (GK, diabetic) and Wistar rats, 3 months old, 160 g ± 10 g, sourced from Rabbitco Pharm (Egypt), housed under pathogen-free conditions. No previous procedures. |
| **9. Experimental procedures** | a. What, how, and with what. b. When/how often. c. Where. d. Why. | **Methods: Animal Model (lines 228–254)** — Ligature-induced periodontitis using sterile 4-0 silk around second molars; checked weekly; 6-week duration; euthanasia by cervical dislocation. Human samples collected once during clinical visit. Purpose: validate molecular findings and histology of inflammation. |
| **10. Results** | a. Summary/descriptive statistics. b. Effect size/confidence intervals. | **Results (lines 267–384)** — Descriptive statistics (mean ± SD, median IQR, p-values). Correlations (Spearman r), AUC values, sensitivity/specificity provided. No explicit confidence intervals. |

| **Item** | **Recommendation** | **Section / Line Number or Explanation** |
| --- | --- | --- |
| **11. Abstract** | Provide summary including objectives, species, strain, sex, key methods, findings, conclusions. | **Abstract (lines 34–58)** — Includes aims, sample numbers (human + rats), major results, and conclusions. Fully compliant. |
| **12. Background** | a. Rationale and context. b. Relevance of model. | **Introduction (lines 69–117)** — Explains bidirectional T2DM-periodontitis link, inflammatory mechanisms, and relevance of NF-κB/miRNA pathways. Animal model justified as mechanistic validation. |
| **13. Objectives** | Clearly describe research question/hypothesis. | **Introduction (lines 103–117)** — Hypothesis: Dysregulation of NFKB1/miRNA axis contributes to inflammatory pathogenesis of diabetic periodontitis. |
| **14. Ethical statement** | Name of ethics committee, licence numbers. | **Ethical Approval (lines 160–168)** — MIU-IRB FWA #00022887, IRB #00010118; Ain Shams University FWA #000017585. Human and animal ethics approvals stated. |
| **15. Housing and husbandry** | Housing, enrichment. | **Methods: Animal Model (lines 211–217)** — Housed in pairs, wire-mesh cages, 21 ± 1 °C, 55 ± 5 % humidity, 12h light/dark, standard diet + water ad libitum. |
| **16. Animal care and monitoring** | a. Pain/distress minimisation. b. Adverse events. c. Humane endpoints. | **Methods: Animal Model (lines 218–254)** — Anaesthesia with xylazine + ketamine for all surgical procedures. No adverse events or humane endpoints beyond euthanasia described. |
| **17. Interpretation / scientific implications** | a. Interpret in light of objectives and literature. b. Limitations and bias. | **Discussion (lines 389–540)** — Results interpreted within diabetic-periodontitis context; detailed limitations (sample size, gender imbalance, cross-sectional design). |
| **18. Generalisability / translation** | Discuss relevance to other species or human biology. | **Discussion (lines 520–543)** — Findings in rats parallel human outcomes; proposed translational diagnostic potential for human T2DM-associated periodontitis. |
| **19. Protocol registration** | Indicate if protocol registered. | **MIU registered** — No statement in manuscript. |
| **20. Data access** | State if/where data available. | **Not reported.** *“Data available upon reasonable request from corresponding author.”* |
| **21. Declaration of interests** | a. Conflicts of interest. b. Funding sources. | **Acknowledgment / Author affiliations (front page & footer)** — No explicit conflict statement; implied none. *“Authors declare no conflicts of interest. This study received no specific grant funding.”* |
